# Albumin tailoring fluorescence and photothermal conversion effect of near-infrared-II fluorophore with aggregation-induced emission characteristics

**DOI:** 10.1038/s41467-019-10056-9

**Published:** 2019-05-17

**Authors:** Shuai Gao, Guoguang Wei, Sihang Zhang, Binbin Zheng, Jiaojiao Xu, Gaoxian Chen, Mingwang Li, Shaoli Song, Wei Fu, Zeyu Xiao, Wei Lu

**Affiliations:** 1 Key Laboratory of Smart Drug Delivery, Ministry of Education, & State Key Laboratory of Molecular Engineering of Polymers, School of Pharmacy & Minhang Hospital, Fudan University, Shanghai, 201203 China; 2 Institute of Molecular Medicine, Clinical and Fundamental Research Center, Renji Hospital, & Department of Pharmacology and Chemical Biology, Shanghai Jiao Tong University School of Medicine, Shanghai, 200127 China; 3 Department of Nuclear Medicine, Shanghai Cancer Center, Fudan University, Shanghai, 200032 China

**Keywords:** Cancer imaging, Cancer therapy, Nanoparticles, Imaging techniques and agents

## Abstract

Fluorophores with donor-acceptor-donor groups with the emission spanning the second near-infrared window (NIR-II) have recently received great attention for biomedical application. Yet, the mechanism underlying the equilibrium between fluorescence (radiative decay) and photothermal effect (non-radiative decay) of these fluorophores remains elusive. Here, we demonstrate that a lipophilic NIR-II fluorophore, BPBBT, possesses both twisted intramolecular charge transfer (TICT) and aggregation-induced emission (AIE) characteristics. Human serum albumin (HSA) binds to BPBBT, which changes the planarity of the fluorophore and restricts its intramolecular rotation. The binding results in alteration to the equilibrium between AIE and TICT state of BPBBT, tailoring its fluorescence and photothermal efficiency. Under the guidance of intraoperative NIR-II fluorescence image, the prepared HSA-bound BPBBT nanoparticles delineate primary orthotopic mouse colon tumor and metastatic lesions with dimensions as small as 0.5 mm × 0.3 mm, and offer photothermal ablation therapy with optimized timing, dosing and area of the laser irradiation.

## Abstract (web summary)

There is a balance between the fluorescence and photothermal properties of fluorescent molecules. Here, the authors report on an NIR-II fluorophore which binds with human serum albumin changing the equilibrium, increasing the photothermal efficiency, and demonstrate application of this for tumour ablation.

## Introduction

Fluorescence imaging in the second near-infrared window (NIR-II, 1000–1700 nm) holds great promise for biomedical application^1,2^. In comparison with NIR-I (700–950 nm) fluorescence imaging, NIR-II imaging has a longer wavelength of emission, lower auto-fluorescence or tissue scattering, deeper tissue penetration, and  higher spatial resolution and contrast, thus dramatically improving imaging quality and signal-to-noise ratio^3,4^. Researches have focused on the development of organic fluorophores with good biocompatibility demonstrating the feasibility of expediting the clinic translation of NIR-II imaging^5–15^. Most of the reported organic fluorophores possess donor–acceptor–donor (D–A–D) structures that lower the band gap allowing the emission to span the NIR-II^5–9^. On the other hand, some D–A–D structured dyes have intense non-radiative decay exhibiting photoacoustic^7,11,12^ or photothermal effect^9,16^. Certain NIR-II fluorophores with D–A–D structures have recently been developed for dual NIR-II imaging and photoacoustic imaging^7^ or dual NIR-II imaging and photothermal therapy^9^. However, the underlying mechanism that determines the equilibrium between fluorescence (radiative decay) and photothermal/photoacoustic effect (non-radiative decay) of these fluorophores remains to be understood.

Qian et al. have reported a lipophilic D–A–D fluorophore BPBBT with a maximum fluorescence emission in NIR-II and an AIE effect^17^. Studies have shown that fluorophores with D–A–D groups have a planar structure in non-polar solvents, producing intense fluorescence due to local excitation (LE)^17–19^. In polar solvents, the intramolecular rotation increases the dihedral angle in the molecule and breaks the planar structure, which reduces radiative transition and greatly weakens fluorescence^19–21^. This phenomenon is referred to as twisted intramolecular charge transfer (TICT). As a result, TICT state enhances non-radioactive transition and the photothermal conversion effect of the molecules^22,23^. Introduction of aggregation-induced emission (AIE) groups (AIEgens) to the structure of D–A–D fluorophores, on the other hand, restricts intramolecular rotation and suppresses TICT, but increases fluorescence when aggregation in poor solvents^19,23–25^. Taken together, TICT state weakens the fluorescence intensity but elevates the photothermal conversion ability of the fluorophores, while AIE state causes enhanced fluorescence intensity but decreased photothermal conversion effect. These phenomena are determined by the nature of intramolecular rotation^19,22,26,27^.

In this work, we discover that BPBBT binds to serum albumin through Van der Waals interactions and hydrogen bonds in high affinity. The specific binding restricts the intramolecular rotation and changes the planarity of the fluorophore. The binding tailors the fluorescence and photothermal conversion properties of BPBBT applicable to intraoperative NIR-II fluorescence image-guided cancer photothermal therapy (PTT). The prepared HSA-bound BPBBT nanoparticles (BPBBT NPs) offer high specific imaging of not only orthotopic mouse colon tumor but also metastatic lesions. Under the guidance of NIR-II fluorescence imaging, BPBBT NPs allow the precisely controlled PTT of both primary and metastatic lesions through optimization of timing, dosing, and area of the laser irradiation, achieving a complete cure of the orthotopic colon tumor in mice.

## Results

### BPBBT with both TICT and AIE characteristics

**Fig. 1:**
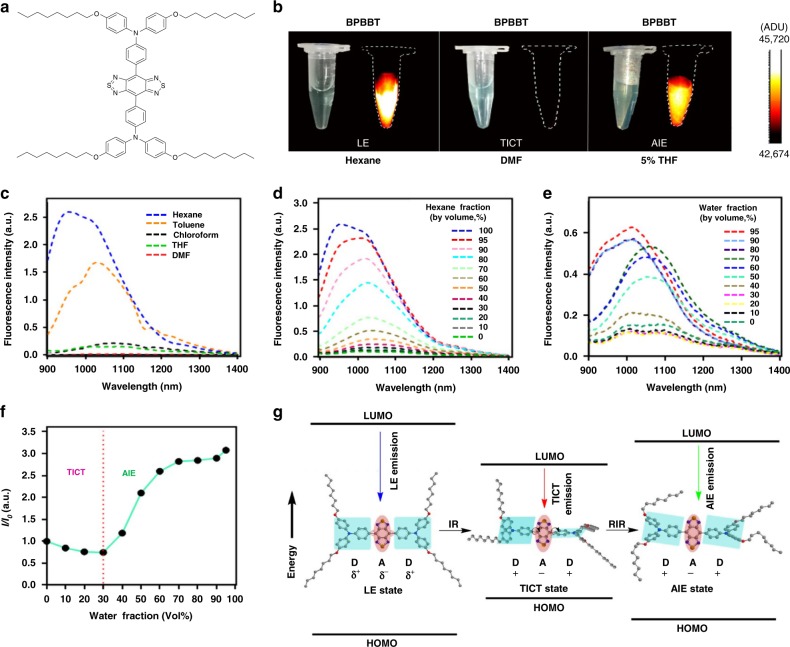
Optical characterization of BPBBT. **a** Chemical structure of BPBBT. **b** Photographs and NIR-II fluorescence images of BPBBT (10 µM) in hexane (non-polar solvent), DMF (polar solvent) or THF containing 95% water (poor solvent for BPBBT) excited at 808 nm (100 mW cm^−2^). LE, locally excited state; TICT, twisted intramolecular charge transfer state; and AIE, aggregation-induced emission state. **c** Fluorescence emission spectra of BPBBT (10 µM) in different solvents after excitation at 831 nm. **d** Fluorescence emission spectra of BPBBT (10 µM) in hexane/THF mixture with hexane fraction from 0 to 100% excited at 831 nm. **e** Fluorescence emission spectra of BPBBT (10 µM) in water/THF mixture with water fraction from 0 to 95% excited at 831 nm. **f** Plot of *I*/*I*_0_ of BPBBT versus water fraction in water/THF mixture. *I* and *I*_0_ represent the fluorescence intensity of BPBBT (10 µM) in water/THF mixture with different water fraction and in THF, respectively. **g** Schematic illustration of the transformation of LE, TICT, and AIE states of BPBBT. D, donor; A, acceptor; IR, intramolecular rotation; RIR, restriction of intramolecular rotation; HOMO, Highest occupied molecular orbital; LUMO, Lowest unoccupied molecular orbital

The hydrophobic BPBBT (Fig. 1a and Supplementary Fig. 1) emitted intense fluorescence in the non-polar solvent, n-hexane (Fig. 1b). Whereas, in the polar solvent, N,N-dimethylformamide (DMF), BPBBT exhibited fluorescence quenching. In a mixture of tetrahydrofuran (THF) and water (5:95, v/v), the fluorescence of BPBBT reappeared though not as high as that in n-hexane. The fluorescence quenching effect on BPBBT was dependent on the polarity of the solvent (Fig. 1c). The emission quantum yield (QY) of BPBBT in solvent decreased as the solvent polarity increased: hexane (8.72%) > toluene (5.56%) > chloroform (0.90%) > THF (0.72%) > DMF (0.42%) (Supplementary Methods and Supplementary Fig. 2a). With the increase of the polarity of the hexane/THF mixture, the fluorescence intensity of BPBBT decreased and the emission peak exhibited red-shift (Fig. 1d). This phenomenon was ascribed to the transition from LE state to TICT state^19^. Addition of water to THF at a water fraction value from 0 to 30% further elevated the solvent polarity and decreased the fluorescence intensity of BPBBT (Fig. 1e and Supplementary Fig. 2b). However, in the mixture with larger fraction of water the fluorescence intensity of BPBBT increased. The highest intensity was achieved in 95% of water. Additionally, the emission spectrum was blue-shifted when water fraction was 80% or above (Fig. 1e) due to the AIE state^19,28^. This result evidenced that the fluorescence intensity of BPBBT was dominated by TICT state at a water fraction value below 30%, but the equilibrium was moved to AIE state when further raising the water fraction (Fig. 1f).

Our findings demonstrated the TICT and AIE states of D–A–D fluorophores^19,23,29^, which was able to be explained using the theory of energy difference between the highest occupied molecular orbital (HOMO) and lowest unoccupied molecular orbital (LUMO) levels (Fig. 1g). From LE state to TICT state, the conformation of BPBBT was twisted, thus elevating the HOMO level and narrowing the band gap for red-shifting the emission spectrum. The reduced florescence intensity was attributed to non-radiative quenching processes^19^. In poor polar solvent such as large fraction of water (≥80%), BPBBT formed nano-aggregation that restricted the intramolecular rotation, leading to blue shift of the emission spectrum in comparison of that of BPBBT in THF and elevation of fluorescence intensity^19^.

### Albumin tailoring the optical properties of BPBBT

**Fig. 2:**
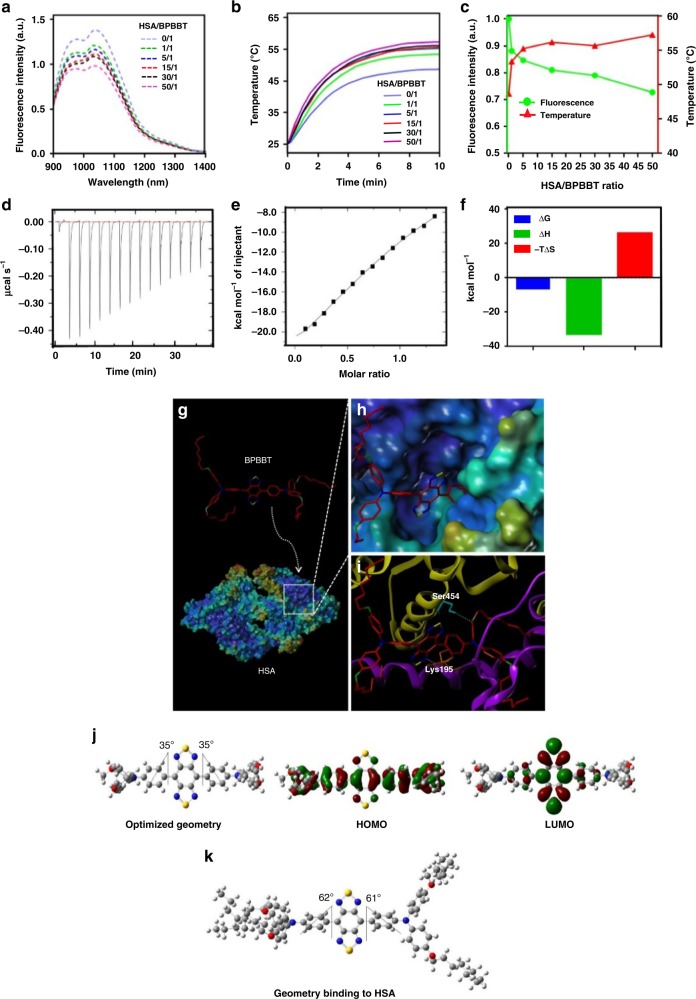
Characterization of HSA/BPBBT complexes. **a** Fluorescence emission spectra of BPBBT (200 µM) in THF:water (5%, v/v) with different HSA-to-BPBBT ratio (w/w) excited at 831 nm. **b** Temperature-time curves of BPBBT (200 µM) in THF:water (5%, v/v) with different HSA-to-BPBBT ratio (w/w) under 808 nm laser irradiation (0.8 W cm^−2^). **c** Plot of *I*/*I*_0_ (green curve) or temperature (red curve) of BPBBT in THF:water (5%, v/v) versus HSA/BPBBT ratio. *I* and *I*_0_ represent the fluorescence intensity of BPBBT calculated from (**a**). Temperature of BPBBT at 10 min in (**b**). **d**–**f** Isothermal titration calorimetry (ITC) analysis of the interaction between HSA and BPBBT. The thermodynamic parameters are determined during every injection of BPBBT into HSA aqueous solution containing 5% THF. **d** Raw data of the integrated heat after correction for heat of dilution. **e** The integrated heat against molar ratio (HSA:BPBBT). The solid line is the fitted curve. **f** The analysis of ITC results from (**d**, **e**). The binding of BPBBT to HSA is exothermic in nature (ΔH < 0) and characterized by a negative entropic change (ΔS < 0). The free energy change of binding is negative (ΔG < 0). **g** Overview of the molecular modeling of BPBBT binding to HSA (PDB code: 5ID7); **h** The active pocket; **i** The inside binding residues, Lys195 (orange) and Ser454 (cyanine). Yellow dashed lines, hydrogen bonds between HSA and BPBBT. **j** Optimized ground-state (S_0_) geometry in the gas phase, and calculated HOMO and LUMO of BPBBT at B3LYP/6-31G(d) level. The dihedral angles are presented in the figures. To reduce the computational requirements, side chains are replaced by methyl groups. **k** Geometry of BPBBT binding to HSA in **g** with dihedral angles presented

**Fig. 3:**
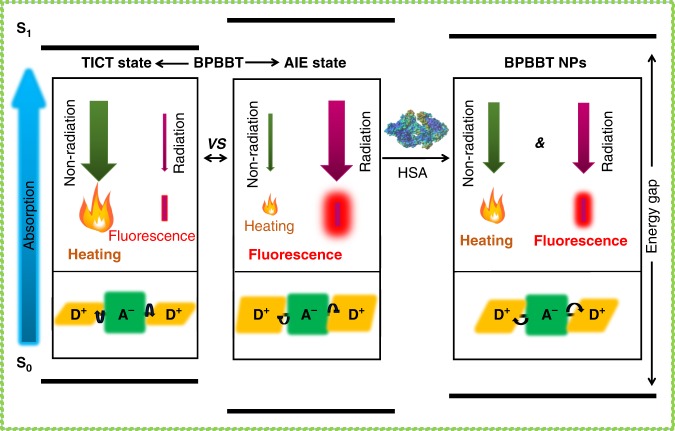
Diagram. HSA altering fluorescence and photothermal efficiency of BPBBT

Since human serum albumin (HSA) was known as an endogenous carrier responsible for transporting lipophilic molecules in the body^30^, we mixed BPBBT with HSA and evaluated the optical property of the HSA/BPBBT complexes. With the increase of HSA ratio in the complexes the fluorescence intensity decreased, while the photothermal conversion effect increased (Fig. 2a–c). Isothermal titration calorimetry (ITC) analysis of the thermodynamic parameters for the binding interaction between HSA and BPBBT illustrated that BPBBT interacted with HSA in a micromolarity range of affinity (Table 1). Figure 2d, e depicted the ITC enthalpogram during the titration of HSA with BPBBT. The negative free energy changes of the binding (ΔG < 0) indicated that the processes were thermodynamically favorable (Fig. 2f). Results from molecular docking and molecular dynamics simulation demonstrated that BPBBT bound to a specific pocket of HSA through Van der Waals interactions and forming hydrogen bonds with Ser454 and Lys195, respectively (Fig. 2g–i). The binding caused conformational constrains of BPBBT and restricted intramolecular rotation. The calculated HOMO, LUMO and dihedral angles between the two investigated aromatic units of the molecule at the optimized ground-state (S_0_) were plotted in Fig. 2j. However, the geometry of BPBBT when binding to HSA displayed larger dihedral angles compared to those of S_0_ (Fig. 2k), suggesting that the binding reduced the planarity of BPBBT. Collectively, HSA specifically bound to BPBBT in high affinity leading to restricted intramolecular rotation but enhanced dihedral angles. The binding reduced the planarity of BPBBT, moving the equilibrium from AIE state toward TICT state. The fluorescence intensity (radiative decay) of BPBBT decreased while the photothermal conversion effect (non-radiative decay) increased when bound to HSA in a dose-dependent manner (Fig. 3).

**Table 1:** Thermodynamic parameters of HSA/BPBBT interaction obtained from ITC measurements

System	∆H (kcal mol^−1^)	∆S (cal mol^−1^ degree^−1^)	∆G (kcal mol^−1^)	*K*_D _(M)	Molar ratio
HSA/BPBBT	−32.64	−87.3	−6.62	(1.46 ± 0.16) × 10^−5^	1.21 ± 0.02

H, enthalpy; S, entropy; G, Gibbs free energy; *K*_D,_ dissociation constant

### Characterization of HSA-bound BPBBT nanoparticles

**Fig. 4:**
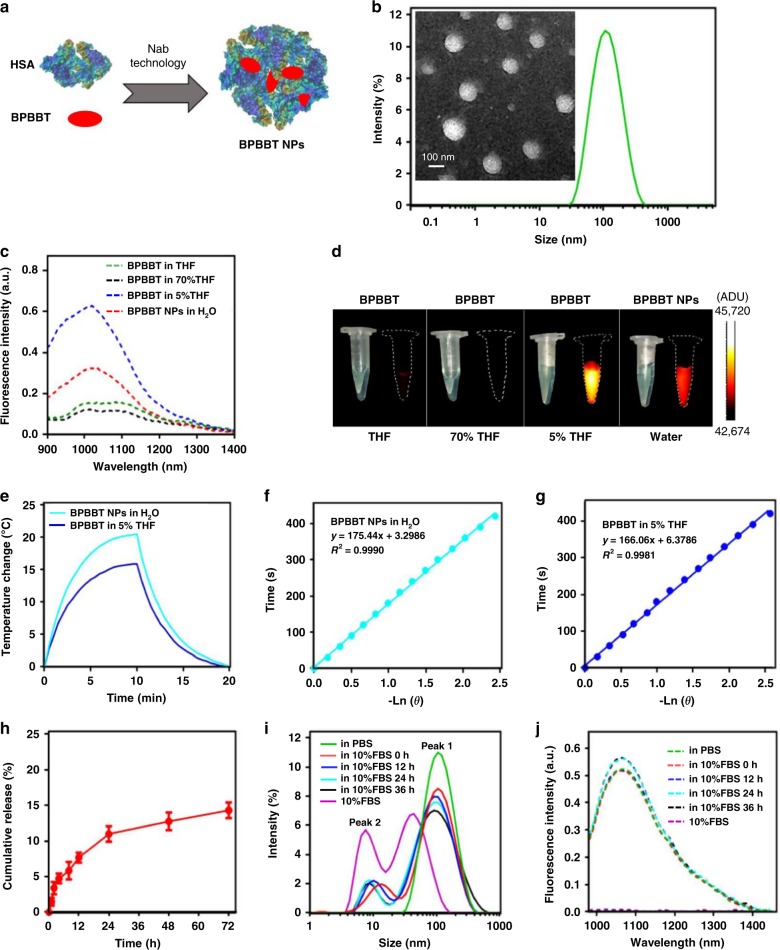
Characterization of BPBBT NPs. **a** Schematic illustration of BPBBT NPs. **b** Representative TEM image and size distribution of BPBBT NPs. **c** Fluorescence emission spectra of BPBBT (10 µM) in THF, 70% THF, 5% THF or BPBBT NPs (10 µM of BPBBT) in water excited at 831 nm. **d** Photographs and NIR-II fluorescence images of the solutions in (**c**). **e** Temperature-time curves of BPBBT NPs (50 µM of BPBBT) in water or BPBBT (50 µM) in 95% water:5% THF under the laser irradiation (808 nm, 1 W cm^−2^) for initial 10 min, followed by 10 min of cooling. **f**, **g** Time constant for heat transfer from the system of BPBBT NPs or BPBBT in 95% water:5% THF is determined to be τ_s_ = 175 s or 166 s by applying the linear time data from the cooling period versus negative natural logarithm of driving force temperature, which is obtained from the cooling stage of panel (**e**). **h** Release profile of BPBBT from BPBBT NPs in 10% FBS. Data are presented as Mean ± S.D. (*n* = 3). **i** Size distribution of 10% FBS, BPBBT NPs in PBS, or BPBBT NPs in 10% FBS at different time points following mixture. Peak 1 from the nanoparticles; Peak 2 from serum. **j** Fluorescence emission spectrum of 10% FBS, BPBBT NPs in PBS, or BPBBT NPs in 10% FBS for different incubation time

**Fig. 5:**
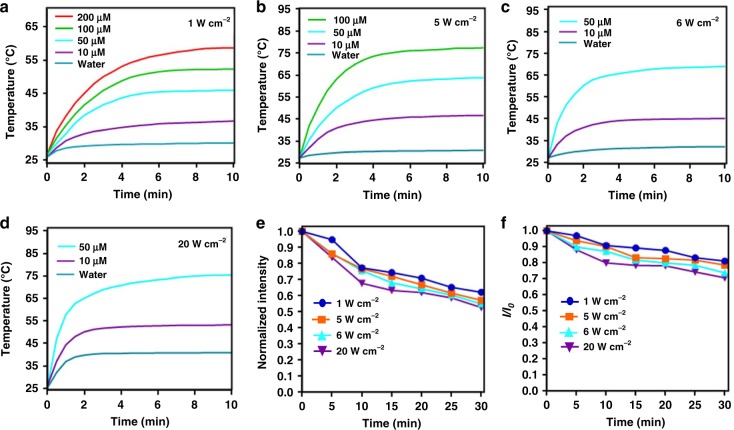
Photothermal conversion behaviors and photostability of BPBBT NPs. **a**–**d** Photothermal effect of BPBBT NPs at different concentrations under an 808-nm light irradiation with different power densities. **e** Normalized fluorescence intensity of BPBBT NPs solutions (50 µM of BPBBT) at the maximum emission wavelength over time following an 808-nm laser irradiation with different power densities. **f** Plot of *I/I*_0_ versus the irradiation time. *I*_0_ and *I* are the maximal NIR absorbance of BPBBT NPs solutions (50 µM of BPBBT) before (0 min) and after laser irradiation, respectively

The high affinity of BPBBT to HSA prompted us to consider using Nab technology^30^ to prepare BPBBT/HSA nanoparticles in order to solubilize the molecule for in vivo application (Fig. 4a). Transmission electron micrograph (TEM) depicted that BPBBT NPs had an average of ~98 nm in diameter, while results from dynamic light scattering (DLS) analysis were a bit larger (~120 nm, Fig. 4b). Immuno-gold staining confirmed the binding of BPBBT NPs to the secreted protein acidic and rich in cysteine (SPARC) (Supplementary Fig. 3), proving the retention of the binding activity of HSA to SPARC following the formulation of BPBBT NPs. The molar extinction coefficient of BPBBT NPs in aqueous solution was 0.9 × 10^4^ M^−1^·cm^−1^ at 808 nm (Supplementary Fig. 4). The fluorescence QY of BPBBT NPs was 1.45%, which was in between that of BPBBT in 5% THF (2.50%) with AIE feature and that in 70% THF (0.43%) with TICT feature (Fig. 4c, d and Supplementary Fig. 2b). On the other hand, the photothermal conversion efficiency of BPBBT NPs (27.5%) was higher than that of BPBBT in 5% THF (22.0%, Fig. 4e–g). These results confirmed that HSA was able to tailor the optical property of BPBBT through nanoformulation. The time-release profile showed that less than 15% of BPBBT was released from BPBBT NPs in 10% fetal bovine serum (FBS) within 72 h (Fig. 4h). DLS analysis illustrated that 10% FBS without addition of the nanoparticles exhibited two size distributions peaked at ~7 nm and ~38 nm, respectively (Fig. 4i, purple). BPBBT NPs in PBS showed a single distribution peaked at ~120 nm (Fig. 4i, green). Immediately after addition of BPBBT NPs to 10% FBS, DLS plot presented two distributions peaked at ~120 nm derived from the nanoparticles (Peak 1) and at ~10 nm from the serum (Peak 2) (Fig. 4i, red). The mixture maintained the same distribution profile within 36 h. Although the height of Peak 1 slightly decreased over time, the ratio of area under curve of Peak 1 to that of Peak 2 did not reduced, indicating that BPBBT NPs were stable in serum for at least 36 h. The fluorescence intensity of BPBBT NPs incubated with 10% FBS remained almost unchanged for 36 h (Fig. 4j). As shown in Fig. 5a–d, the in vitro photothermal effect of BPBBT NPs in water was dependent on the nanoparticle concentration and the laser power density. BPBBT NPs maintained 84–95% of fluorescence intensity and 88–97% of the maximal NIR absorbance following the laser treatment with the power densities up to 20 W cm^−2^ for 5 min, indicating that BPBBT NPs had a relatively good photostability (Fig. 5e, f, and Supplementary Fig. 5). The cellular uptake of BPBBT NPs by CT26-Luc mouse colon cancer cells was in a concentration-dependent manner (Supplementary Fig. 6a). The in vitro photothermal ablation effect of BPBBT NPs on CT26-Luc cells was also dependent on the nanoparticle concentration incubated and the laser dose applied (Supplementary Fig. 6b). BPBBT NPs exhibited minimized cytotoxicity to mouse embryonic fibroblasts NIH 3T3 cells following 24-h incubation (Supplementary Fig. 6c). Systemic administration of BPBBT NPs at a dose of 20 mg kg^−1^ of BPBBT did not cause changes of the majority of blood chemistry and hematologic parameters except for a reversible change of blood urea nitrogen (BUN) within one-month post-injection (Supplementary Table 1). The hematoxylin and eosin (H&E) staining did not exhibit significant toxicity in major organs (Supplementary Fig. 7). The fluorophore was observed to be excreted from the body through feces (Supplementary Fig. 8). These results suggested that BPBBT NPs had good biocompatibility without significant toxicity at a dose of 20 mg kg^−1^ of BPBBT.

### Intraoperative NIR-II imaging of tumor with BPBBT NPs

**Fig. 6:**
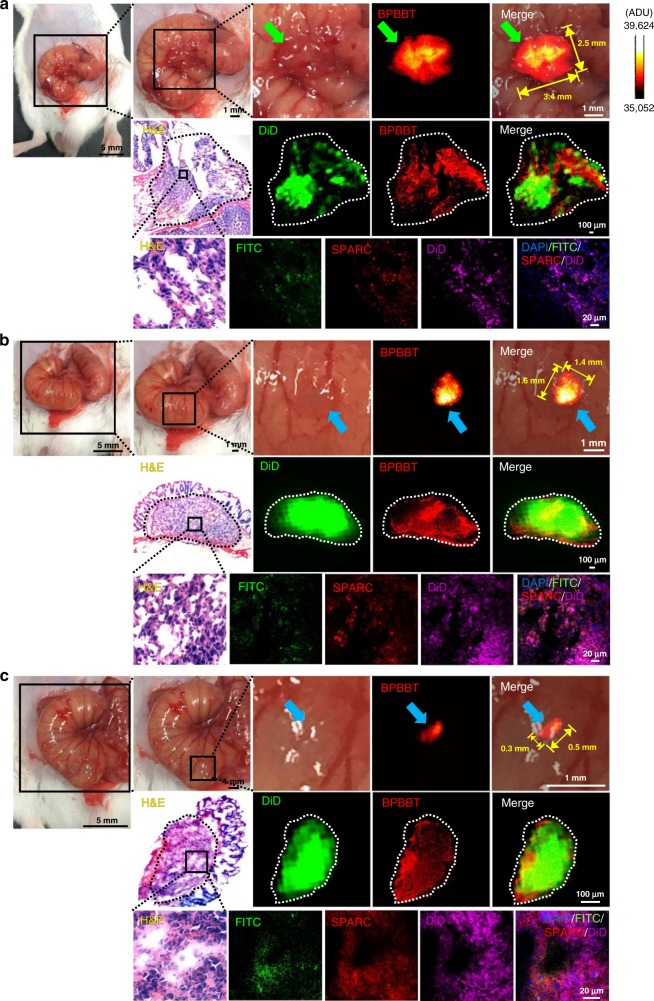
Intraoperative NIR-II imaging of orthotopic mouse colon tumor by BPBBT NPs. BALB/c mouse 1 bearing DiD-labeled CT26-Luc tumor was i.v. injected with BPBBT NPs (20 mg kg^−1^). After 30 h, an incision was made in the abdominal skin of mouse under the anesthetic condition to expose cecum and the adjacent area for NIR-II imaging. **a** NIR-II live imaging (upper) and histologic analysis (middle and lower) of primary tumor in the cecum of the tumor-bearing mouse. Green arrows, primary tumor lesion. **b, c** NIR-II live imaging (upper) and histologic analysis (middle and lower) of metastatic tumor foci in 1.6 mm × 1.4 mm size (**b**) or in 0.5 mm × 0.3 mm size (**c**) in the cecum. Blue arrows, metastatic lesions. Dashed circles, tumor area. See Supplementary Figs 11 and 12 for the imaging of mice 2 and 3, Supplementary Movie 1 for the imaging of mouse 4

We next established both subcutaneous and orthotopic CT26-Luc colon cancer model in mice to evaluate if the optical properties of BPBBT NPs were eligible for NIR-II fluorescence image-guided cancer PTT in vivo^31,32^. NIR-II live imaging of mice bearing subcutaneous tumor revealed that the fluorescence reached the maximal intensity in tumor at 30 h following intravenous (i.v.) injection of BPBBT NPs (Supplementary Fig. 9). Biodistribution of BPBBT in CT26-Luc orthotopic tumor-bearing mice also confirmed that the highest BPBBT distribution in tumor tissues was achieved at 30 h post-injection of BPBBT NPs (Supplementary Fig. 10). The amount of BPBBT distributed in the primary tumor and in the metastatic tumor was 5.8-fold and 4.0-fold of that in cecum at 30 h post-injection, respectively. Therefore, this time point was determined for NIR-II fluorescence image-guided PTT in the orthotopic model. To track the tumor cells in the orthotopic model, we labeled the tumor cells with 1,1′-dioctadecyl-3,3,3′,3′-tetramethylindodicarbocyanine, 4-chlorobenzenesulfonate salt (DiD) before the inoculation. Besides the primary lesion, we found that the inoculated tumor cells spontaneously developed at least one site of metastatic lesions in cecum in all the investigated mice at 4 d post-inoculation (*n* = 40). In addition, we labeled BPBBT NPs with fluorescein isothiocyanate (FITC), BPBBT NPs-FITC, to track the distribution of the nanoparticles. At 30 h following injection of BPBBT NPs-FITC, the tumor-bearing mice received an incision of the abdominal skin under anesthetic condition with cecum exposed. Intraoperative NIR-II imaging of the cecum and the adjacent area delineated a primary tumor lesion with dimensions of 3.4 mm × 2.5 mm (Fig. 6a, upper row, green arrows). Histologic analysis confirmed that the fluorescence of BPBBT colocalized with that of the DiD-labeled tumor cells (Fig. 6a, middle row, circled area). In addition, the adjacent normal tissue did not display the NIR-II fluorescence. Immunofluorescence micrograph demonstrated that the FITC-labeled nanoparticles colocalized with SPARC in tumor (Fig. 6a, lower row), supporting the tumor targeting effect of BPBBT NPs through the albumin-SPARC binding^33,34^. Remarkably, the NIR-II imaging identified the two metastatic lesions of the same mouse with dimensions of 1.6 mm × 1.4 mm and 0.5 mm × 0.3 mm, respectively (Fig. 6b, c). It should be emphasized that these metastatic foci were invisible under bright-field scope (Fig. 6b, c, upper rows, blue arrows). The accuracy of the NIR-II fluorescence in the metastatic tumor was also confirmed by histologic analysis (Fig. 6b, c, middle and lower rows). Significantly, the NIR-II fluorescence was only detected in the metastatic tumor but not the adjacent normal cecal tissue (Fig. 6b, c, middle rows). We repeated the experiment in additional three tumor-bearing mice to confirm this observation (Supplementary Figs 11 and 12, and Supplementary Movie 1). These results evidenced that the i.v. administered BPBBT NPs-mediated intraoperative NIR-II imaging accurately identified both primary and metastatic tumor of sizes as small as 0.5 mm × 0.3 mm in the orthotopic colon cancer model.

**Fig. 7:**
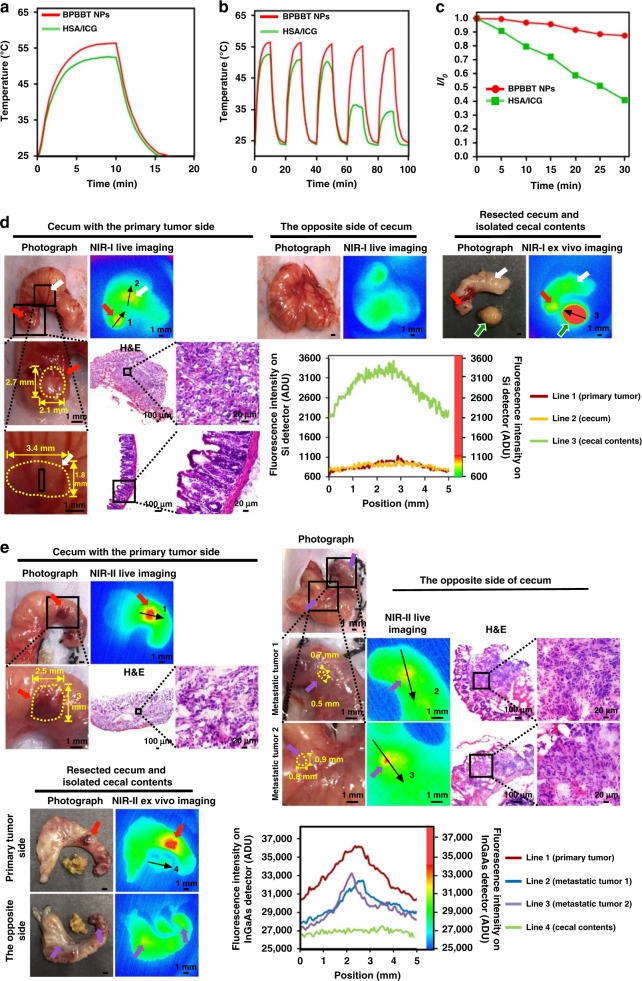
Superior optical properties of BPBBT NPs to that of HSA/ICG complexes. **a** Temperature-time curves of BPBBT NPs (200 µM of BPBBT) or HSA/ICG complexes (200 µM of ICG) in PBS. Each sample was irradiated with laser (808 nm, 0.8 W cm^−2^) for the first 10 min. **b** Stability of photothermal conversion effect of BPBBT NPs or HSA/ICG complexes during five cycles of heating-cooling processes in (**a**). **c** Plot of *I/I*_0_ versus irradiation time. *I* and *I*_0_ are the maximal NIR absorbance of BPBBT NPs (200 µM of BPBBT) and HSA/ICG complexes (200 µM of ICG) in PBS solutions before (0 min) or after laser irradiation (808 nm, 0.8 W cm^−2^), respectively. **d** NIR-I imaging of the mouse orthotopic CT26-Luc colon cancer by HSA/ICG complexes (20 mg kg^−1^) at 30 h following injection. Left and upper middle, intraoperative imaging of the exposed cecum on the side of primary tumor (red arrows) and the opposite side, respectively. Upper right, ex vivo imaging of the resected cecum on the side of the primary tumor and the isolated cecal contents (green arrows). White arrows, normal cecum with false positive NIR-I fluorescence signals. Lower right, cross-sectional fluorescence intensity profiles of the selected area along the direction of black arrows in the fluorescence images. **e** NIR-II imaging of the mouse orthotopic CT26-Luc colon cancer by BPBBT NPs (20 mg kg^−1^) at 30 h following the injection. Upper left and upper right, intraoperative imaging of cecum on the side of primary tumor (red arrows) and the opposite side, respectively. Purple arrows, metastatic tumor. Lower left, ex vivo imaging of the resected cecum and the isolated cecal contents on both sides. Lower right, cross-sectional fluorescence intensity profiles of the selected area along the direction of black arrows in the fluorescence images. An 808 nm laser (100 mW cm^−2^) was used for the imaging in (**d**, **e**)

We then compared the optical property of BPBBT NPs with that of the HSA/indocyanine green (ICG) complexes (Supplementary Fig. 13) that were reported for NIR-I image-guided PTT^35^. Following irradiation with 808 nm laser (0.8 W cm^−2^) for 10 min, the temperature of BPBBT NPs solution (200 µM of BPBBT) increased to 56 °C, while the temperature of HSA/ICG complexes solution (200 µM of ICG) reached to 53 °C (Fig. 7a). Moreover, following three cycles of the laser irradiation, the photothermal conversion effect of HSA/ICG complexes drastically decreased (Fig. 7b). By contrast, the photothermal conversion effect of BPBBT NPs remained nearly unchanged (Fig. 7b). After laser irradiation for 30 min, the light absorbance of HSA/ICG complexes dropped to ~40%, while that of BPBBT NPs remained ~90% of their original absorbance (Fig. 7c and Supplementary Fig. 14). Compared to HSA/ICG complexes, BPBBT NPs offered higher photothermal conversion effect and photostability.

Following the injection of HSA/ICG complexes (20 mg kg^−1^ of ICG), intraoperative NIR-I imaging with a silicon detector (10 ms; 830–900 nm) delineated a primary tumor with dimensions of 2.7 mm × 2.1 mm (Fig. 7d, left panel, red arrows). Nevertheless, a false positive signal near the tumor was observed with dimensions of 3.4 mm × 1.8 mm (white arrows), which was normal cecum evidenced by the H&E staining. No metastatic lesions were detected in the images of both sides of the cecum (Fig. 7d, left and upper middle panels). Imaging of the resected cecum following removal of cecal contents confirmed the positive signal at the primary tumor site (Fig. 7d, upper right panel, red arrows), while the false positive signal observed in the live image disappeared (white arrow). Strikingly, the removed cecal contents displayed extremely high NIR-I fluorescence, which was ~3 folds as high as that of the tumor (Fig. 7d, upper right panel, green arrows; lower right panel). These results supported that the HSA/ICG complexes-mediated NIR-I imaging offered sensitivity only enough to detect primary tumor but not metastatic lesions in the orthotopic colon cancer model. Moreover, the NIR-I imaging displayed false positive signals due to the intense fluorescence of the cecal contents.

By contrast, intraoperative NIR-II imaging of tumor-bearing mice receiving BPBBT NPs with an InGaAs detector (100 ms, >1000 nm) demonstrated sharp contrasts of both primary and metastatic tumor (Fig. 7e, upper left panel and upper right panel). The fluorescence intensity of the removed cecal contents was significantly lower than that of either primary or metastatic tumor (Fig. 7e, lower left panel and lower right panel). These results proved that BPBBT NPs-mediated NIR-II imaging provided more accurate and sensitive detection of the orthotopic colon tumor than HSA/ICG complexes-mediated NIR-I imaging.

### Intraoperative NIR-II image-guided photothermal therapy

**Fig. 8:**
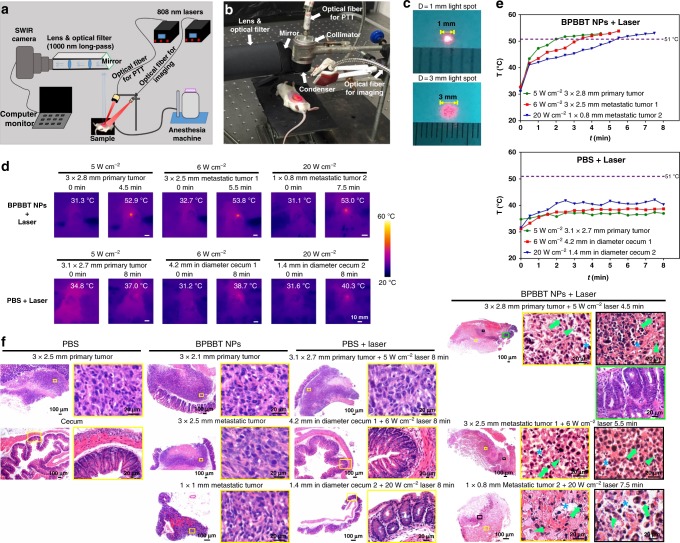
Validation of intraoperative NIR-II image-guided cancer PTT with BPBBT NPs. **a** Schematic illustration of home-made system for NIR-II image-guided PTT of tumor in mice. **b** Photograph of NIR-II image-guided PTT of BALB/c mice bearing orthotopic CT26 colon cancer under anesthetic condition. **c** Measurement of laser spot adjusted to 1 mm or 3 mm in diameter for PTT. **d** Infrared (IR) thermal images before (0 min) and at the end of PTT of the tumor-bearing mice at 30 h following i.v. injection with BPBBT NPs (20 mg kg^−1^) or PBS. In the BPBBT NPs-treated mice, primary and metastatic tumor was identified under NIR-II imaging followed by PTT with the indicated laser power density. In the PBS group, primary tumor was visualized by eye. Due to the invisible metastatic lesions, areas of cecum next to the primary lesion were randomly selected for PTT. **e** Temperature of the irradiated area-time curves from the IR thermal imaging of (**d**). **f** H&E staining of tumor or normal cecum of mice at 24 h following different treatment. In the BPBBT NPs-treated mice, primary and metastatic tumor was visualized under NIR-II imaging. In the PBS group, primary tumor was visualized by eye, while the metastatic lesions were invisible. Area of normal cecum was randomly selected. Tumor necrosis was characterized by extensive pyknosis (arrows), karyolysis (arrowheads), cytoplasmic acidophilia, and degradation of the extracellular matrix of tumor (asterisks)

We next set up a home-made NIR-II image-guided PTT system (Fig. 8a, b). The tumor-bearing mice injected with BPBBT NPs firstly received the intraoperative NIR-II imaging for the identification of both primary and metastatic tumor as well as the tumor sizes. The tumor was then illuminated by a red-light guide beam from the diode laser through an optical fiber connected to a collimator and a condenser at the distal end. The illumination area of the laser was precisely controlled to just completely cover the exposed tumor through adjusting the distance between the condenser and the object (Fig. 8c). Literature reported that tissue thermal damage depends on the exposure time linearly and on the temperature elevation exponentially^36,37^. Thermal doses of 120–240 min at 43 °C (*t*_43_) induce significant tissue necrosis^36^. Based on the plot of temperature-time thresholds of acute irreversible thermal damage, 100 s is required for the threshold damage at 51 °C (*t*_51_)^37,38^. Accordingly, we used the infrared thermal imaging to monitor the temperature of tumor during PTT to optimize the laser power and irradiation time. Our results illustrated that for the primary tumor with dimensions of 3 mm × 2.8 mm of mice injected with BPBBT NPs, *t*_51_ required an 808 nm laser illumination for 4.5 min with 5 W cm^−2^ of power density and 4.1 mm in diameter of laser spot (Fig. 8d, e, and Supplementary Fig. 15). For primary tumor of the similar size of mice receiving PBS, such laser dose only increased the temperature to 37 °C. For the metastatic tumor with dimensions of 3 mm × 2.5 mm, *t*_51_ required the laser dose of 6 W cm^−2^, the laser spot of 3.9 mm in diameter, and 5.5 min of the irradiation time. For the smaller metastatic lesion with dimensions of 1 mm × 0.8 mm, *t*_51_ required the laser dose of 20 W cm^−2^, the laser spot of 1.3 mm in diameter, and 7.5 min of the irradiation time. In PBS-treated mice, since the metastatic tumor was not detected by eye, normal cecum was tested for thermal response. The laser at 20 W cm^−2^ (1.4 mm in diameter of spot) only increased the temperature of cecum to 40–42 °C (Fig. 8d, e, and Supplementary Fig. 15). H&E staining confirmed a complete necrosis of both primary and metastatic tumor of mice receiving BPBBT NPs plus the optimized PTT, which was characterized by extensive cytoplasmic acidophilia, pyknosis, karyolysis, and degradation of the extracellular matrix of tumor (Fig. 8f, BPBBT NPs + Laser, yellow and black boxes). Whereas, the histology of the adjacent normal cecum remained unchanged (Fig. 8f, BPBBT NPs + Laser, green box). In tumor-bearing mice receiving PBS, the three laser doses did not cause damage to either primary tumor or normal cecum (Fig. 8f, PBS + Laser). These results validated the optimal laser doses for both primary and metastatic tumor in different sizes that generated a complete thermal necrosis of the tumor but least damage to the surrounding normal tissue.

**Fig. 9:**
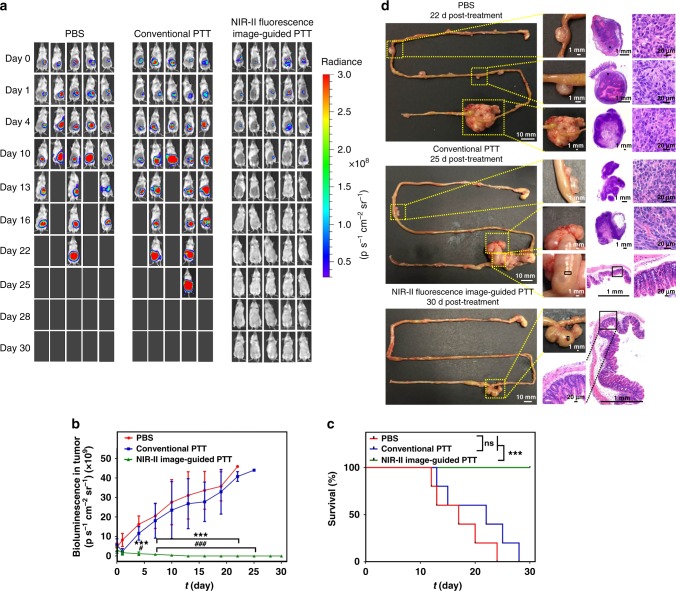
Therapeutic efficacy of intraoperative NIR-II image-guided PTT by BPBBT NPs. **a** The in vivo bioluminescence imaging of BALB/c mice bearing orthotopic CT26 colon cancer before (day 0) or after different treatment (*n* = 5). In the group of NIR-II fluorescence image-guided PTT, mice received i.v. injection of BPBBT NPs (20 mg kg^−1^). After 30 h, primary and metastatic tumor was visualized under NIR-II imaging followed by PTT with the optimized doses according to the tumor sizes. In the group of conventional PTT, mice were i.v. injected with BPBBT NPs (20 mg kg^−1^). After 30 h, only primary lesion visualized by eye received PTT with the optimized doses according to the tumor sizes. In both treated groups, the cecum was repositioned following PTT. The skin was closed with suture. In PBS group, mice received i.v. injection of PBS without further treatment. **b** Tumor growth curves of mice bearing orthotopic CT26-Luc colon cancer following different treatment measured by the bioluminescence. Data are presented as Mean ± S.D. (*n* = 5). Statistical significance was calculated via two-way analysis of variance (ANOVA) with a Tukey post-hoc test. ***p < 0.001 between the NIR-II image-guided PTT group and PBS group. ^#^*p* < 0.05, ^###^*p* < 0.001 between the NIR-II image-guided PTT group and conventional PTT group. **c** Kaplan–Meier survival curve of mice bearing orthotopic CT26-Luc colon cancer following different treatment (*n* = 5). Statistical significance was calculated via the log-rank Mantel–Cox test. ***p < 0.001 between the two compared groups. ns, no significant difference between the two compared groups. **d** Photographs of the resected gastrointestinal tract and histological examination of tumor or cecum of mice at the end of each treatment with H&E staining. See Supplementary Fig. 16 for the other four mice in the NIR-II image-guided PTT group

Mice bearing CT26-Luc orthotopic tumor receiving intraoperative NIR-II image-guided PTT with the optimized laser doses presented a complete cure (5 out of 5) without recurrence in 30 d (Fig. 9a–c). Histological examination revealed that the cecum appeared normal and no metastatic lesions were detected throughout the gastro-intestine tract (Fig. 9d and Supplementary Fig. 16). On the contrary, the mice receiving conventional PTT, i.e., PTT of tumor detected by eye, had severe tumor recurrence based on the bioluminescence imaging with the medium survival time of 22 d. Considerable tumor metastases were observed in the conventional PTT group (Fig. 9d).

## Discussion

We utilize the theory of restricted intramolecular rotation^19,22–24,39^ to explain the mechanism underlying the equilibrium between TICT state and AIE state of the NIR-II D–A–D fluorophore with AIE characteristics. Understanding such mechanism prompts to explore strategy, that is to use albumin to restrict the intramolecular rotation and change the dihedral angles of BPBBT through Van der Waals interactions and formation of hydrogen bonds, to tailor the equilibrium of BPBBT between AIE state and TICT state. Addition of HSA to the lipophilic BPBBT decreases its radiative decay but enhances non-radiative decay in a dose-dependent manner. Through the preparation of the albumin-bound BPBBT nanoparticles, we successfully tune the fluorescence and the photothermal conversion effect of BPBBT eligible for dual fluorescence imaging and PTT. The theory of restricted intramolecular rotation may also apply to explain the mechanism of the recently reported AIE Dots for dual fluorescence and photoacoustic imaging^7^. When encapsulated in a polymer matrix, the intramolecular rotation of the lipophilic NIR-II fluorophore with AIEgens would be restricted, resulting in large NIR absorptivity and an emission maximum near 1000 nm^7^. The mechanism will help the design of other nano-complexes to adjust the optical properties of other NIR-II D–A–D fluorophore with AIE characteristics.

Successful cancer PTT relies on accurate and high-resolution imaging techniques to detect invisible tumor lesions that are hardly identified by human eye^40–43^. Moreover, the real-time imaging monitors the distribution of the photothermal agents, which is allowed to determine the optimal timing and dosing of laser irradiation^1,44,45^. Organic NIR-I fluorescent dye such as ICG exhibits combined properties of cancer NIR-I imaging and photothermal ablation as well as advantages of little-to-no toxicity and metabolizability^46,47^. However, the fluorescence emission wavelength of ICG is in NIR-I region, where tissue penetration of light is suboptimal^48,49^. Besides, ICG is limited to fluorescence bleaching and thermal instability.

By contrast, BPBBT NPs offer an NIR-II fluorescence imaging with high resolution and specificity that detect invisible tumor lesions with minimal metastasis of size as small as 0.5 mm × 0.3 mm (Fig. 6c and Supplementary Fig. 12b). This advantage remarkably improves the accuracy of PTT and avoids missing small lesions. Comparatively, such small lesions cannot be detected under the HSA/ICG complexes-mediated NIR-I fluorescence imaging (Fig. 7d). Additionally, HSA/ICG complexes generate false positive signals due to the intense NIR-I fluorescence of the cecal contents (Fig. 7d). Compared with HSA/ICG complexes, BPBBT NPs exhibit higher photothermal conversion effect and photostability (Fig. 7a–c). The NIR-II fluorescence of BPBBT NPs allows monitoring the distribution of BPBBT in real-time for optimizing timing and dosing of laser irradiation (Fig. 8). The phototheranostic agents, BPBBT NPs, offer optimal PTT to be confined to the tumor lesions under the guidance of NIR-II imaging, which significantly reduces phototoxicity to the surrounding normal tissue. The partially tumor cell-killing effect of BPBBT NPs may be attributed to the possible photosensitization activity of BPBBT, although no current literature has reported reactive oxygen species (ROS) generated by BPBBT or other fluorophore with a benzobisthiadiazole core. The ROS generation by BPBBT will be investigated in future study.

In summary, we have demonstrated that protein such as albumin tailored the equilibrium between AIE state and TICT state of the lipophilic NIR-II D–A–D fluorophore with AIE characteristics. The preparation of albumin-bound fluorophore nanoparticles has been successfully used for intraoperative NIR-II fluorescence imaging of orthotopic mouse colon tumor and metastatic lesions with dimensions as small as 0.5 mm × 0.3 mm. The intense photothermal conversion effect of BPBBT NPs offered a complete cure of mice bearing colon cancer following the optimized PTT under the guidance of NIR-II fluorescence imaging. The BPBBT NPs held great promise for NIR-II fluorescence image-guided cancer PTT, especially for colon cancer phototheranostics.

## Methods

### General measurements

BPBBT, 4,8-Bis[4-(*N*,*N*-bis (4-octyloxyphenyl)amino) phenyl]benzo[1,2- *c*:4,5-*c*′] bis ([1,2,5] thiadiazole), was synthesized according to previous publication with minor modifications (Supplementary Fig. 1)^17^. ^1^H NMR spectrum of BPBBT in CDCl_3_ solution was recorded on a Bruker AV400 at 400 MHz (Germany) using tetramethylsilane (*δ* = 0 ppm) as the internal standard. ^13^C spectrum was obtained on the same instrument at 100 MHz using CDCl_3_ (*δ* = 77 ppm) as the internal reference. Ultraviolet-visible-near-infrared (UV-VIS-NIR) absorption spectra were recorded on a UV-2401PC UV/vis spectrophotometer (Shimadzu, Japan). DLS measurement was performed using a DLS analyzer (Malvern 3000, UK). All samples (0.1 mg mL^−1^) were measured at 25 °C and at a scattering angle of 173^o^. The nanoparticles were visualized under a transmission electron microscopy (FEI Tecnai G^2^ 20 TWIN, LaB6, USA) operated at 200 kV with negative staining (2% phosphotungstic acid). The NIR-II fluorescence emission spectra were measured using a fluorescence spectrometer (PTI QM40, USA).

### Isothermal titration calorimetry (ITC)

The thermodynamic parameters for HSA/BPBBT interactions were determined by a MicroCal ITC200 system (Malvern, UK) at 25 °C. HSA in the cell was prepared in water with 5% THF at a concentration of 20 µM, which was 10-fold lower than the corresponding concentration of BPBBT in the syringe. A total of 15 injections were used in this experiment. The volume of the solution was 0.4 μL for the first injection and 2.5 μL for the remaining 14 injections. The separation between injections was 150 s. This experiment was repeated six times.

### Molecular calculation of protein binding capacity

The X-ray structure of HSA was downloaded from the website (PDB code: 5ID7). The structure was prepared by SYBYL-6.9 (including residue repair and energy minimization). Method: Powell; Termination: gradient with 0.05 kcal mol^−1^; Max iterations: 10,000; Force field: MMFF94s; Charges: MMFF94.

### Density functional theory calculations

All the calculations were performed using the Gaussian 09 software. To reduce the computational cost, alkyl substituent groups on the fluorophores were replaced by methyl groups. The ground-state (S_0_) geometry of the simplified structure BPBBT was optimized at the B3LYP/6-31G(d) level in the gas phase. The HOMO and LUMO, absorption excitation energies of the molecule were obtained at the B3LYP/6-31 G(d) level based on its optimized S_0_ geometry.

### In vitro NIR-II fluorescence imaging

The NIR-II fluorescence imaging of BPBBT (10 μM) in hexane, toluene, THF, chloroform, DMF or THF containing different volumes of deionized water or PBS was measured using a liquid-nitrogen-cooled short wave infrared camera with a 640 × 512-pixel, two-dimensional InGaAs array detector (ITR, 100 ms, Xenics Cougar, Belgium). The excitation light was provided by a fiber-coupled 808 nm laser (Cnilaser, China) with a power density of 100 mW cm^−2^. The emission light was filtered using 1000 nm long-pass filter (Thorlabs).

### Preparation of BPBBT NPs and HSA/ICG complexes

BPBBT NPs were prepared using nanoparticle albumin-bound (Nab) technology. Briefly, 450 mg of HSA was dissolved with 30 mL of water to a final concentration of 1.5% (w/v). BPBBT (30 mg) was dissolved in 0.68 mL of chloroform/ethanol (15/85, v/v). The HSA solution was mixed with BPBBT solution and the mixture was homogenized (Nano debee homogenizer, USA) under 20,000 psi for nine cycles. The resulting colloid suspension was rotary evaporated at 25 °C for 5 min under reduced pressure. Then, the nanoparticles were filtered through a 0.22 µm microporous membrane filter followed by lyophilization. For the preparation of HSA/ICG complexes, 10 mg of ICG (Meilunbio, China) was resuspended in 11.4 mL of sterile water. Then, this mixture was transferred to a 1.5 mL of 20% HSA solution to yield stock solutions of ICG in HSA (HSA/ICG complexes) at a final concentration of 1 mM. The complexes were also filtered through a 0.22 µm microporous membrane filter.

### Photothermal stability tests

The aqueous solutions of BPBBT NPs or HSA/ICG complexes were irradiated under an 808 nm laser (0.8, 1, 5, 6 or 20 W cm^−2^). Fluorescence emission spectra and absorption spectra of BPBBT NPs in aqueous solutions after irradiated with an 808 nm laser with different power densities were measured at different time points. For anti-photobleaching study, the temperatures of the sample solutions were recorded during five cycles of heating and cooling processes. During one heating-cooling cycle, the samples were irradiated with the NIR laser for the first 10 min to reach to a steady state. Following removal of the laser, the samples were allowed to cool down naturally to the ambient temperature in 10 min. In another experiment, BPBBT in 5% THF, HSA/BPBBT complexes, aqueous solutions of BPBBT NPs or HSA/ICG complexes were exposed to an 808 nm laser (0.8 W cm^−2^) for 10 min.

### Drug release and serum stability test

In vitro release of BPBBT from BPBBT NPs was performed as follows: 3 mL of BPBBT NPs (1.0 µM) was sealed in a dialysis bag (MWCO 3500 Da) and immersed in 97 mL PBS containing 10% (v/v) fetal bovine serum (FBS) at 37 °C at 100 rpm. Sample (1.0 mL) was withdrawn from the medium at designated time intervals and replaced with the same volume of fresh medium. Each sample was mixed with 1.5 mL of THF, vortexed for 3 min. The supernatant was assayed using a fluorescence spectrometer (PTI QM40, USA). For the in vitro stability test: BPBBT NPs were added to 10% FBS (w/v) at 37 °C to give a final concentration of 0.6 mg mL^−1^. DLS analysis was performed at different time points following the mixture. Their fluorescence emission spectra were also measured using a fluorescence spectrometer (PTI QM40, USA) at the same time.

### Establishment of subcutaneous or orthotopic CT26-Luc colon tumor model

All the following animal procedures were in agreement with the guidelines of and approved by the Institutional Animal Care and Use Committee (IACUC) of Fudan University School of Pharmacy. BALB/c mice (male, 6–8 weeks) were purchased from Shanghai Linchang Biological Technology Co.Ltd. For the subcutaneous tumor model, CT26-Luc cells (~2 × 10^6^, Imanis Life Sciences, CT26.WT-Fluc-Neo) suspended in 50 µL of PBS were subcutaneously injected into the right flank of each mouse. For the orthotopic CT26-Luc tumor model, mice were anesthetized and immobilized on a plate. The abdominal skin was sanitized using 0.5% (w/v) of iodophor. A small midline incision (3–5 mm) was cut in the skin of lower abdomen to expose cecum. CT26-Luc cells (~2 × 10^6^) in 25 μL of PBS were injected into the cecal wall. The injected cecum was then put back into the abdominal cavity. The wound was closed using biodegradable suture. The growth of colon tumor foci was monitored using IVIS Spectrum Imaging System (Caliper, PerkinElmer, USA) to measure the bioluminescence through the intraperitoneal injection of D-luciferin (15 mg mL^−1^, 200 µL).

### In vivo NIR-II imaging or NIR-I imaging

For the subcutaneous tumor model, at 10 d following the tumor cell inoculation, the mice were i.v. injected with BPBBT NPs in PBS solution (20 mg kg^−1^). All images were collected at predetermined time points using a home-built NIR-II imaging system with the liquid-nitrogen-cooled InGaAs camera. The excitation light (100 mW cm^−2^) was provided by an optical fiber-coupled 808 nm laser. The emission light passed through a magnification lens set (Olympus) and 1000 nm long-pass filter (Thorlabs), which was collected by the camera.

For the orthotopic tumor model, 3 d after the tumor cell inoculation, BPBBT NPs containing 20 mg kg^−1^ of BPBBT were i.v. injected. At 30 h following the injection, the tumor-bearing mice received an incision of the abdominal skin under anesthetic condition. The cecum was exposed for NIR-II imaging using the same procedure as that for the subcutaneous tumor model.

For NIR-I imaging, the orthotopic tumor model received i.v. injection of HSA/ICG complexes containing 20 mg kg^−1^ of ICG. The NIR-I imaging was conducted at 30 h following the injection. A 2048 × 2048-pixel, water-cooled sCOMS camera with silicon detector (PCO.edge 4.2, Germany) was used to replace the InGaAs camera. An 830 nm dichroic beam splitter (Semrock, USA) was used to replace the 1000 nm long-pass filter.

### FITC-labeled BPBBT NPs

BPBBT NPs were suspended in 0.1 M sodium carbonate buffer to reach a final concentration of 2 mg mL^−1^. FTIC (Meilun, China) in DMSO solution (1 mg mL^−1^) was added into the protein solution at a volume ratio of 1:20. After incubated in dark for 8 h, the labeling reaction was terminated by adding NH_4_Cl. The unconjugated FITC was separated from the conjugates by PD-10 desalting column (GE Healthcare, USA).

### Colocalization study

CT26-Luc cancer cells were labeled by DiD cell-labeling solution (Life Technologies, USA) according to the protocol. The DiD-labeled CT26-Luc cells were injected into the cecal wall of BALB/c mice according to the above method. Three days after the inoculation, the mice were i.v. injected with BPBBT NPs-FITC (20 mg kg^−1^). Primary and metastatic tumor foci were detected and resected under NIR-II imaging guidance via the home-built NIR-II imaging system at 30 h after injection. The resected tissues were embedded in OCT (Sakura Finetek, Japan) for cryosection. Tissue sections of 30 μm thickness were visualized with Odyssey Infrared Imaging System (Li-Cor, USA) for DiD. The same samples were examined under a home-built imaging system with the InGaAs camera for BPBBT. The adjacent sections of 8 µm thickness were stained with H&E or stained with 4′,6-diamidino-2-phenylindole (DAPI) and rabbit anti-mouse SPARC (D10F10) monoclonal antibodies (1:1000, CST, USA, #8725). The secondary antibody was Alexa Fluor 555-conjugated goat anti-rabbit IgG (1:600, Invitrogen, USA, #A21429). The slices were mounted and observed by a Zeiss LSM 710 confocal microscope (Oberkochen, Germany).

### NIR-II fluorescence image-guided PTT

The NIR-II image-guided PTT device was built based on the Cougar camera. The excitation was provided by an 808 nm diode laser through an optical fiber with a power density of 100 mW cm^−2^. Fluorescence signals were collected by passing through a lens set and the 1000 nm long-pass filter. Another 808 nm diode laser was used for PTT treatment through an optical fiber with collimator and condenser at the distal end. By adjusting the distance between the object and the condenser, the size of laser light spot was accurately controlled. Three days after tumor inoculation, the tumor-bearing mice were randomly divided into three groups (*n* = 5): mice receiving BPBBT NPs and NIR-II fluorescence image-guided PTT (Group A), mice receiving BPBBT NPs and PTT without the NIR-II imaging guidance (Group B) and PBS control group (Group C). The mice in Group A or Group B were i.v. injected with BPBBT NPs (20 mg kg^−1^) at 30 h before the PTT treatment. The abdominal skin was sliced to expose cecum under the anesthetic condition with isoflurane. In Group A, primary and metastatic tumor foci were identified via NIR-II imaging. The size of light spot was adjusted according to the actual size of tumor foci so as to just completely cover the tumor. Primary tumor foci were exposed under the laser spot with the power density of 5 W cm^−2^. Large metastatic tumor foci (major axis > 1 mm) were exposed under the laser light spot with the power density of 6 W cm^−2^. Small metastatic tumor foci (major axis ≤ 1 mm) were exposed under the laser light spot with the power density of 20 W cm^−2^. The temperatures in the tumor region were recorded with an infrared camera thermographic system. The irradiation time varied from 4.5 min to 8 min so as to ensure the temperature of tumor foci maintained over 51 °C for at least 2 min (*t*_51_). In Group B, since only primary tumor foci were visualized by eyes, the corresponding tumor foci were exposed under the laser light spot with the power density of 5 W cm^−2^. The size of the spot light and the irradiation time were adjusted according to the actual size and temperature of tumor foci, respectively. In Group C, the mice only received i.v. injection of PBS.

### Histologic analysis

The mice were divided into four groups at 3 d after tumor inoculation: Group A, mice were i.v. injected with 200 μL of PBS. Group B, mice were i.v. injected with BPBBT NPs in PBS solution (20 mg kg^−1^). Group C, mice were i.v. injected 200 μL of PBS and received PTT at 30 h post-injection. Group D, mice were i.v. injected with BPBBT NPs (20 mg kg^−1^) and received PTT at 30 h post-injection. In Group A, primary tumor and normal cecum was resected at 54 h after PBS injection. In Group B, primary and metastatic tumor foci were identified via NIR-II fluorescence imaging system and resected under the NIR-II fluorescence imaging guidance. In Group C, primary tumor foci were irradiated with an 808 nm laser (5 W cm^−2^) for 4.5 min. Two areas of cecum were randomly selected to be irradiated with the same laser dose as that used in PTT of the large and small metastatic tumor foci in above treatment, respectively. The primary tumor and exposed cecum were resected at 24 h post-treatment. In Group D, the treated primary and metastatic tumor foci were resected at 24 h post-treatment. All the resected tissues were fixed in formalin and embedded in paraffin, sectioned, stained with H&E.

### Statistical analysis

Statistical analysis was performed using GraphPad Prism 6 software (GraphPad). Data are presented as the Mean ± S.D. for all results. Statistical significance was determined by two-way analysis of variance (ANOVA) with a Tukey post-hoc test for multiple comparisons. Survival was analyzed using Kaplan–Meier survival curves. The curves were compared with the log-rank Mantel–Cox test. *p* < 0.05 was considered significant.

### Reporting summary

Further information on research design is available in the Nature Research Reporting Summary linked to this article.

## Supplementary information

Supplementary Information

Reporting Summary

Description of Additional Supplementary Files

Supplementary Movie 1

Source Data

## Data Availability

The data that support the findings of this study are available within the paper (and its Supplementary Information files) and from the corresponding author upon reasonable request. The source data underlying Figs. 1c–f, 2a–c, 4b–j, 5a–f, 7a–e, 8e, 9b, c and Table 1 and Supplementary Figs 2a, b, 4, 5a–d, 6a, c, 8b, 9b, 10a, b and 13b and Supplementary Table 1 are provided as a Source Data file.
