# Supplementary material for: Albumin tailoring fluorescence and photothermal conversion effect of near-infrared-II fluorophore with aggregation-induced emission characteristics
Source: Nat Commun. 2019 May 17;10:2206. doi: 10.1038/s41467-019-10056-9 (PMC6525245; doi:10.1038/s41467-019-10056-9)
Supplement: Supplementary file 1 — Supplementary Information [file 41467_2019_10056_MOESM1_ESM.pdf]

Supplementary Information

Albumin tailoring fluorescence and photothermal conversion effect of near-infrared-II fluorophore with aggregation-induced emission characteristics

Gao et al.

Supplementary methods

Calculation of molar extinction coefficient: The Lambert Beer's law was used to calculate the molar extinction coefficient of BPBBT (10 μM) in THF or BPBBT NPs (10 μM of BPBBT) aqueous solution at 808 nm.[1]

Calculation of the photothermal conversion efficiency: According to Roper's report, the photothermal conversion efficiency (η) is expressed as follows:[2]

$$\eta = \frac{hS(T_{Max}-T_S)-Q_C}{I\ (1-\ 10^{-A_{808}})} \qquad (1)$$

Where h is heat transfer coefficient; S is the surface area of the container; the Q_C represents heat dissipated from light absorbed by the quartz sample cell itself, measured 24.2 mW using a quartz cuvette cell containing pure water; T_{Max} is the maximum system temperature, T_S is ambient temperature of the surroundings, and the (T_{Max}-T_S) of BPBBT in 5% THF and BPBBT NPs aqueous solution is 15.9 °C and 20.5 °C according to Figure 4, respectively; I is 1.0 W cm^{-2}; A_{808} is the absorbance of BPBBT in 5% THF (0.251) or BPBBT NPs aqueous solution (0.258) at 808 nm.

Calculation of fluorescence quantum yield (QY): The NIR-II fluorescence QY was calculated according to the previously reported method.[3] Briefly, a series of dilutions of IR-26 (BOCSCI Inc.) as the reference, BPBBT or BPBBT NPs in different solvent were prepared. The fluorescent emission spectra from 1000 to 1450 nm were measured by a fluorescence spectrometer (PTI QM40, USA) in a 1 cm quartz cuvette under the excitation of 808 nm. Absorbance of the above samples was measured by a UV-2401PC UV/vis spectrophotometer (Shimadzu, Japan). The integrated emission intensity was plotted against the absorbance and fitted into a linear function with a slope. The fluorescence QY was calculated as follows:

$$QY_{sample} = QY_{ref} \cdot \frac{slope_{sample}}{slope_{ref}} \cdot \left(\frac{n_{samlpe}}{n_{ref}}\right)^2 \quad (2)$$

Where QY_{ref} of IR-26 in dichloroethane is 0.5%, and n_{sample} and n_{ref} represent the refractive index of BPBBT, BPBBT NPs or IR26 in different solvents.

Immunogold staining: A mixture of BPBBT NPs (3 mg mL^{-1}, 40 μL) and recombinant human SPARC proteins with His tag (0.5 mg mL^{-1}, 10 μL, Sino Biological Inc., #10929-H08H) were stirred at 120 rpm at room temperature away from light for 4 h. The gold nanoparticle-conjugated mouse anti-His tag IgG (15-nm diameter of gold, 1:1, HepengBio, #C030304) was added and stirred for another 4 h under the same condition. Free proteins or antibodies were removed by passing through a Sepharose CL-4B column. The labeled particles were negatively stained with 2% phosphotungstic acid and then examined on an FEI Tecnai G^2 20 TWIN electron microscope (200 kV).

Cellular uptake of BPBBT NPs: CT26-Luc cells were seeded into 6-well plates with RPMI-1640 plus 10% FBS at a density of 5×10^5 cells per well for 24 h. The medium was replaced with RPMI-1640 without FBS containing BPBBT NPs (50, 100 or 200 μg mL^{-1} of BPBBT). After 2-h incubation, the cells were washed three times with cold PBS and collected using cell scrapers. After ultrasonication, the protein content was measured by a BCA protein assay. BPBBT was extracted using toluene followed by quantification on a fluorescence spectrometer (PTI QM40, USA).

***In vitro* photothermal ablation effect:** CT26-Luc cells were incubated with different concentrations of BPBBT NPs. After 2 h, the supernatant was removed and the cells were irradiated for 5 min using an 808 nm laser at 3, 5 and 7 W cm^{-2}, respectively. The laser light covered the central area of the microplate well. After the laser irradiation, the cells were

resupplied with RPMI-1640 containing 10% FBS and incubated at 37 ºC for another 12 h. The cells were then washed with PBS and stained with calcein AM and ethidium homodimer-1. The cells were visualized under a fluorescence microscope (Leica DMI 4000 B).

***In vitro* cytotoxicity:** NIH 3T3 cells (the American Type Culture Collection, CRL-1658) were used for the *in vitro* cytotoxicity evaluation of BPBBT NPs via methyl thiazolyl tetrazolium (MTT) assay. The cells (8×10^3 per well) were cultured in 96-well microplates for 48 h before the experiment. The cells were incubated with a series of concentrations of BPBBT NPs for 24 h. Subsequently, 20 μL of MTT solution (5 mg mL^{-1}) was added to each well. After 2-h incubation, the supernatant was replaced with 150 μL of dimethyl sulfoxide (DMSO) to dissolve crystals. The absorbance was measured at 570 nm using a Bio-Rad 550 microplate reader.

***In vivo* biodistribution analysis**: CT26-Luc orthotopic tumor-bearing mice were i.v. injected with BPBBT NPs (20 mg kg^{-1}) and euthanized at 1, 12, 30 or 48 h post-injection ($n = 5$). Blood and major organs were collected and weighted. Liver, kidney, brain, cecum or cecal contents was mixed with PBS (three times of the tissue weight). Heart, spleen or lung was mixed with 1 mL of PBS. The mixture was homogenized by a homogenizer (Jinxin, China) for 1 min under ice bath. The homogenates of primary tumor foci and metastatic tumor foci were prepared by addition of 300 μL of PBS and homogenized by an ultrasonic cell crusher (Scientz, China) for 20 s under ice bath. To extract BPBBT from different samples, 400 μL of toluene was added into 100 μL of the tissue homogenate or plasma sample. The mixture was emulsified by ultrasonic cell crusher (Scientz, China) for 20 s under ice bath. After centrifugation at 15,000 rpm for 10 min, the supernatant layer was transferred to quartz cuvette cells for measurement

the fluorescence intensity of BPBBT under the fluorescence spectrometer (PTI QM40, USA). The blank tissue samples were added with different BPBBT NPs standard solution, homogenized, and extracted using the same protocol for the validation test.

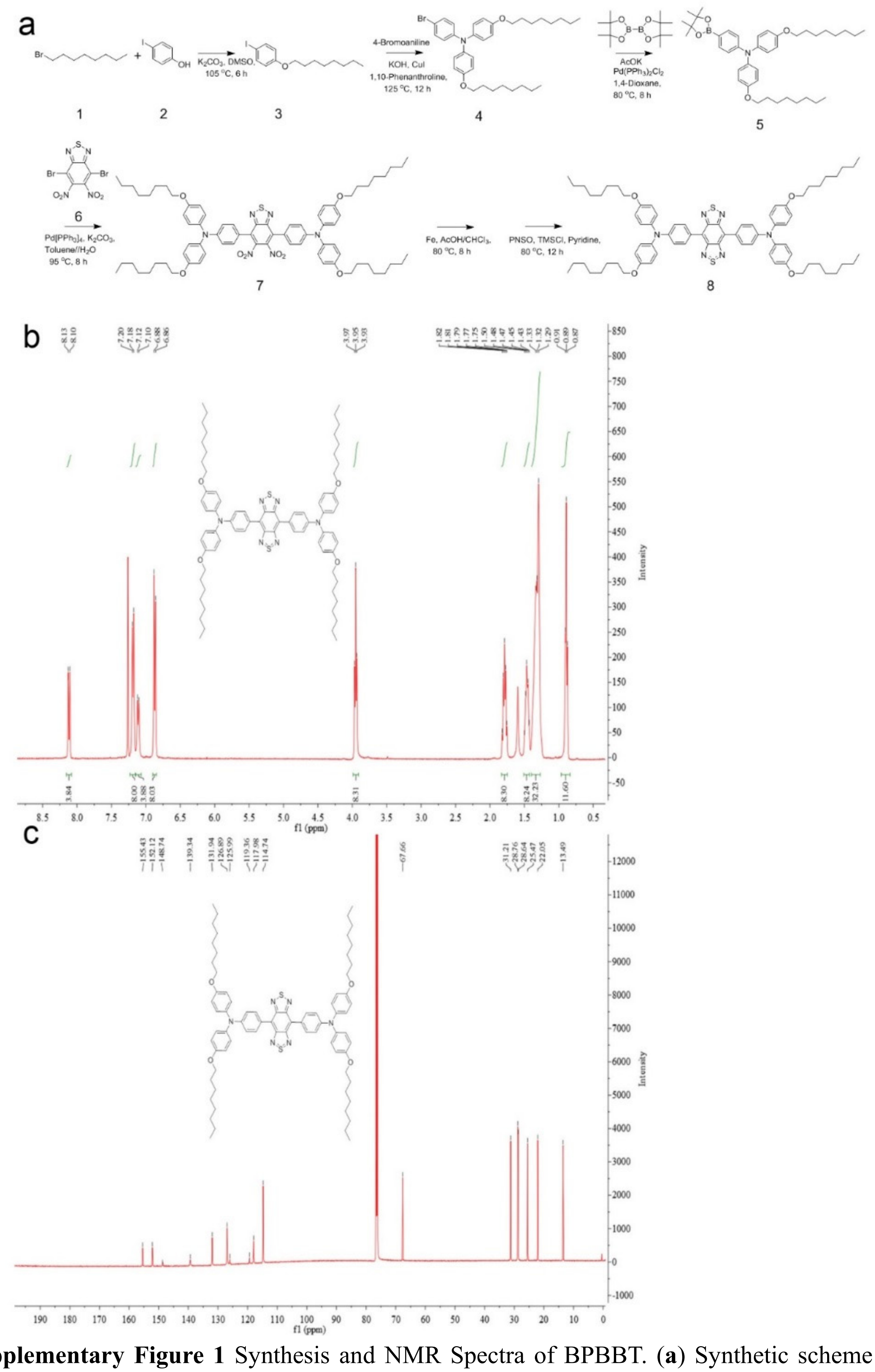

Supplementary Figure 1 Synthesis and NMR Spectra of BPBBT. (**a**) Synthetic scheme of BPBBT. (**b**), ^{1}H NMR (400 MHz, $CDCl_3$) δ 8.12 (d, J = 8.5 Hz, 4H), 7.19 (d, J = 8.3 Hz, 8H), 7.12 (d, J = 8.5 Hz, 4H), 6.87 (d, J = 8.5 Hz, 8H), 3.95 (t, J = 6.5 Hz, 8H), 1.84 – 1.75 (m, 8H), 1.51 – 1.43 (m, 8H), 1.40 – 1.27 (m, 32H), 0.89 (t, J = 6.3 Hz, 12H). (**c**), ^{13}C NMR (100 MHz, $CDCl_3$) δ 131.94, 126.89 , 117.98 , 114.74 , 67.66 , 31.21 , 28.70, 25.47, 22.05, 13.49.

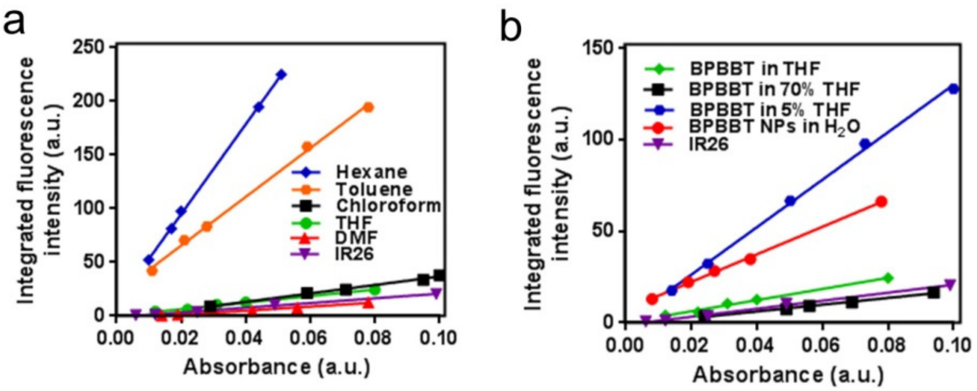

Supplementary Figure 2 Calculation of fluorescence quantum yield (QY). (**a**), Integrated emission intensities of IR-26 or BPBBT in different solvent as a function of 808 nm absorbance. The emission QY of BPBBT in solvent decreased as the solvent polarity increased: hexane (8.72%) > toluene (5.56%) > chloroform (0.90%) > THF (0.72%) > DMF (0.42%). (**b**), Integrated emission intensities of IR26, BPBBT in THF, 70% THF, or 5% THF, or BPBBT NPs in water as a function of 808 nm absorbance. The emission QY of BPBBT in THF, 70% THF, 5% THF and BPBBT NPs in water was 0.72%, 0.43%, 2.50% and 1.45%, respectively. QY of BPBBT in different solvent was calculated based on the reference IR-26 in dichloroethane, whose QY is 0.5% according to the literature.[3]

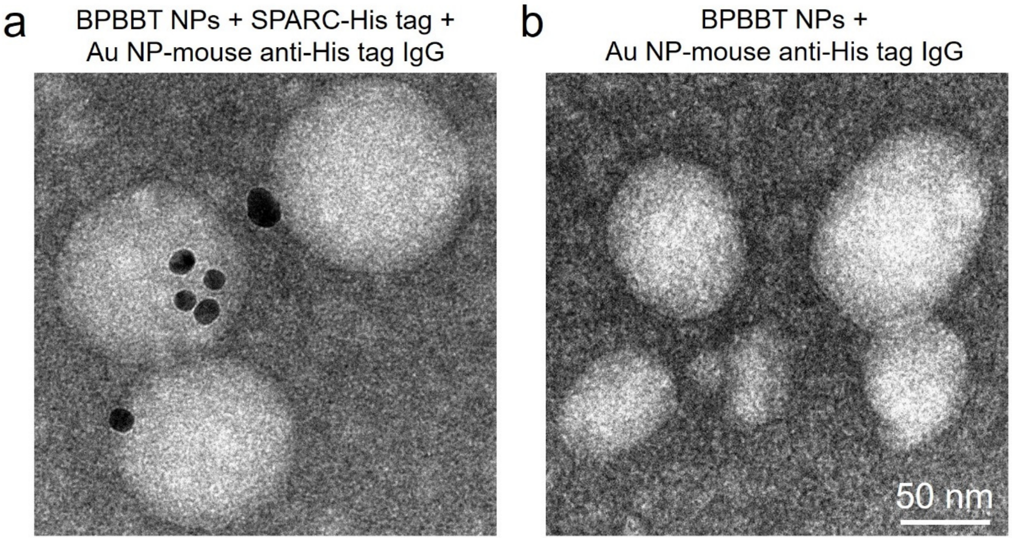

Supplementary Figure 3 TEM images of BPBBT NPs following the immunogold staining. (**a**), BPBBT NPs were incubated with SPARC with His tag, followed by incubation with gold nanoparticle (Au NP)-conjugated mouse anti-His tag IgG. (**b**), BPBBT NPs were only incubated with Au NP-conjugated mouse anti-His tag IgG (Control).

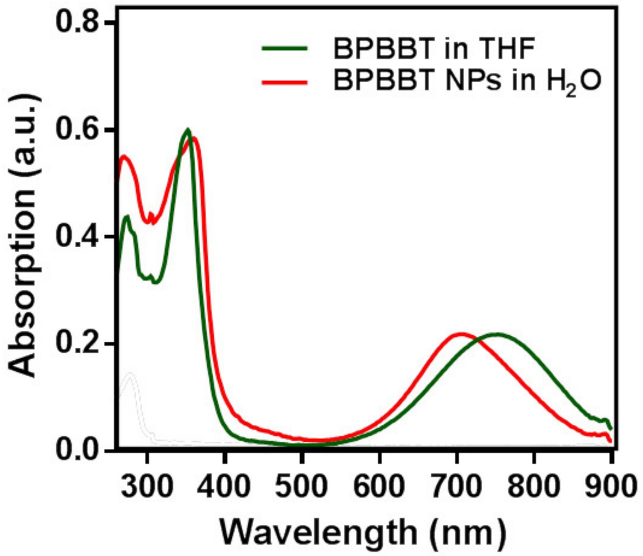

Supplementary Figure 4 UV-Visible absorption spectra of BPBBT or BPBBT NPs (10 μM of BPBBT). The molar extinction coefficient of BPBBT NPs in aqueous solution and BPBBT in THF at 808 nm was calculated 0.9×10^4 $M^{-1}\cdot cm^{-1}$ and 1.7×10^4 $M^{-1}\cdot cm^{-1}$, respectively.

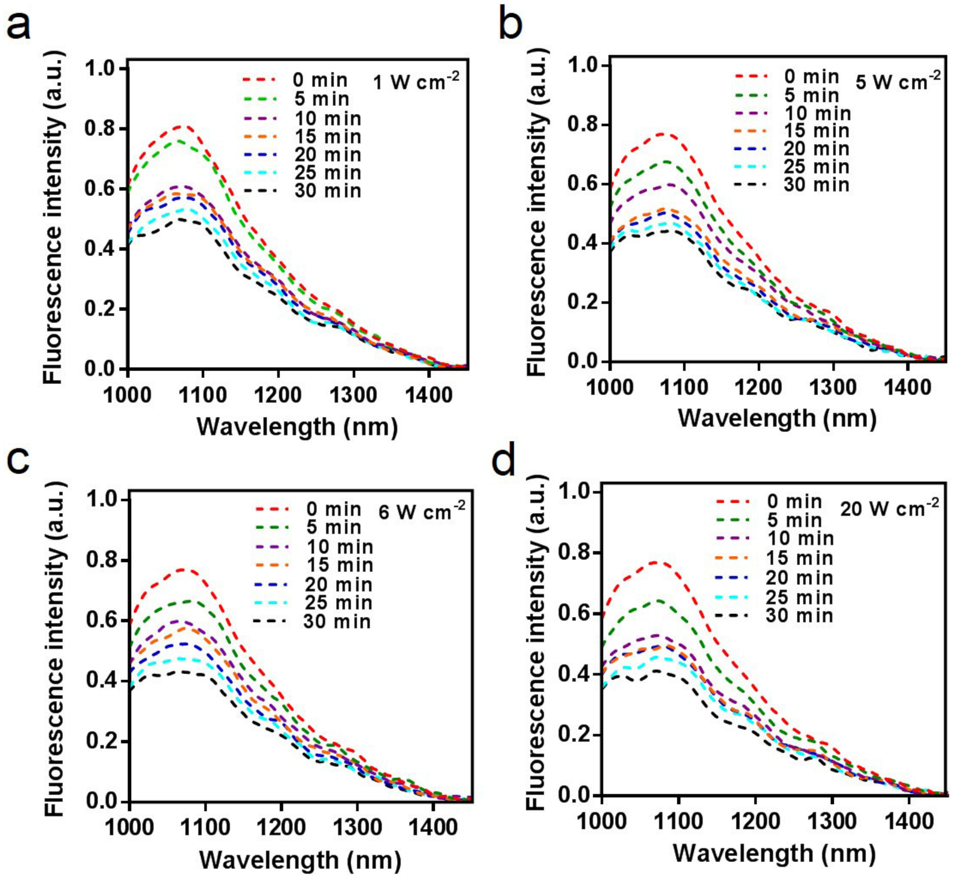

Supplementary Figure 5 Fluorescence emission spectra of BPBBT NPs (50 μM of BPBBT) in aqueous solutions after irradiation using an 808-nm laser at 1 W cm^{-2} (**a**), 5 W cm^{-2} (**b**), 6 W cm^{-2} (**c**) or 20 W cm^{-2} (**d**) for 0-30 min.

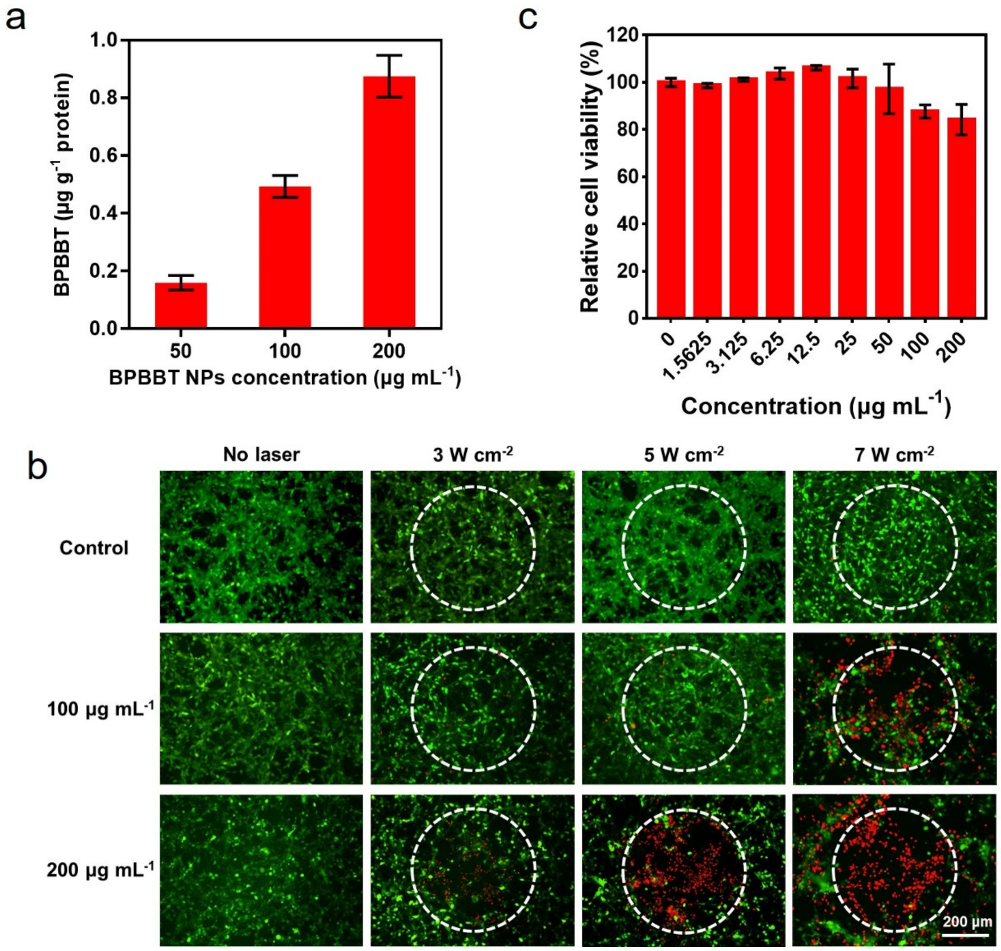

Supplementary Figure 6 (**a**), Uptake of BPBBT NPs by CT26-Luc cells after incubation at different concentrations for 2 h (n = 4). (**b**), Representative fluorescence micrographs of CT26-Luc cells incubated with BPBBT NPs (100 or 200 μg mL^{-1} of BPBBT) for 2 h followed by irradiation using an 808-nm laser with different power densities for 5 min. The cells were stained with LIVE/DEAD® viability/cytotoxicity kit and observed under a fluorescence microscope. Green, live cells stained with calcein AM; Red, dead cells stained with ethidium homodimer-1. Circles, the laser-irradiated area. (**c**), Cell viability of NIH 3T3 cells after incubation with BPBBT NPs at various concentrations for 24 h (n = 3). Data are presented as Mean ± S.D.

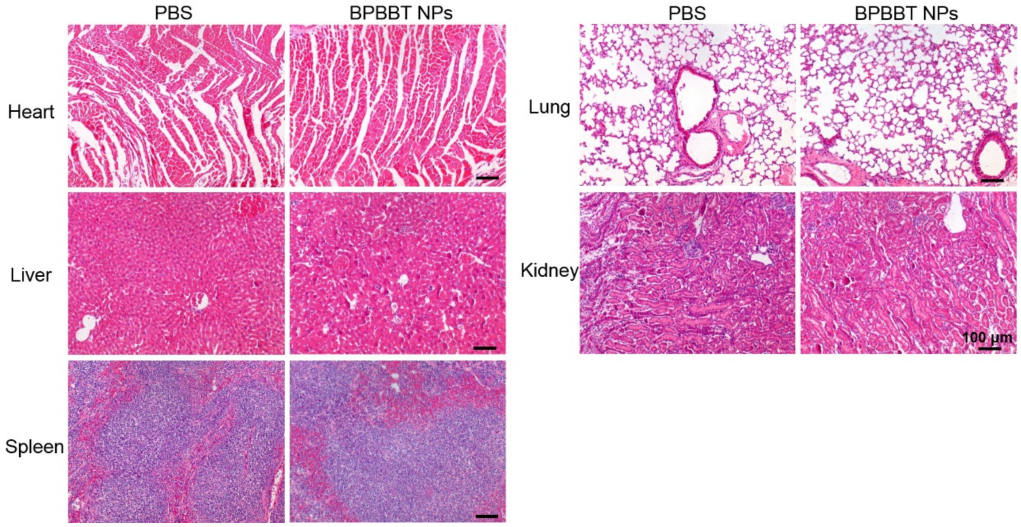

Supplementary Figure 7 Histologic analysis of major organs of healthy mice at 24 h post-injection of BPBBT NPs (20 mg kg^{-1}) or PBS through H&E staining.

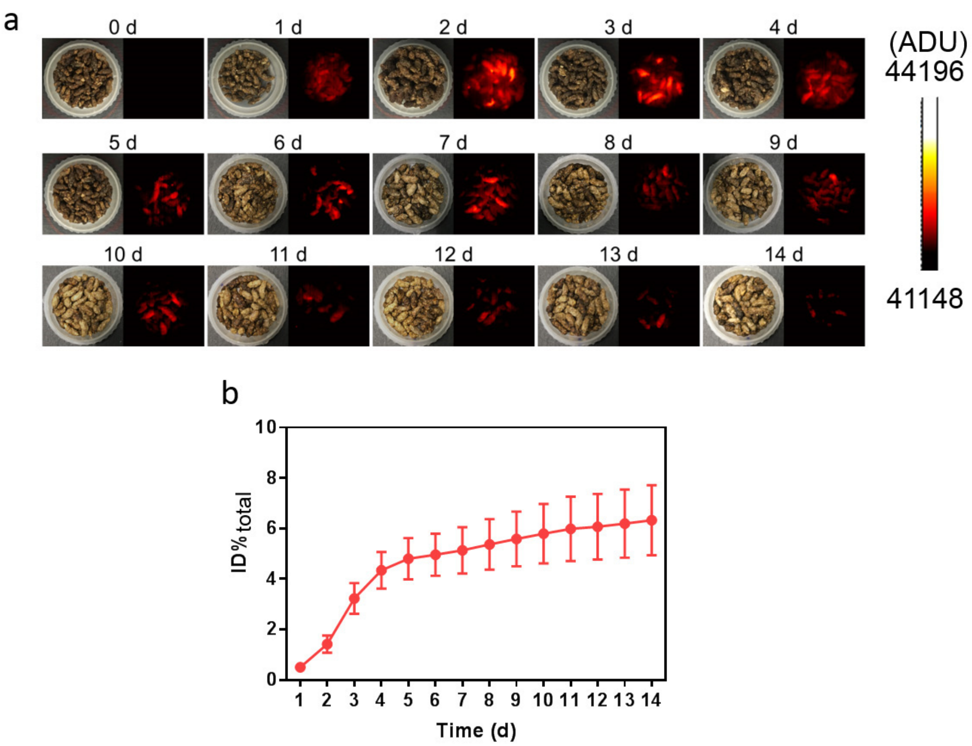

Supplementary Figure 8 Excretion of BPBBT from mice. (**a**), Photographs (left) and the corresponding NIR-II fluorescence images (right) of the collected feces samples at different time after i.v. injection of BPBBT NPs (20 mg kg^{-1}) over 2 weeks. (**b**), Quantitative analysis of BPBBT excreted from feces in **a**. $ID\%_{total}$, Cumulative percentage of the injected doses. Data are presented as Mean ± S.D. (n = 6). Each sample was added with 4 times of THF in volume, milled, vortexed and sonicated for 15 min. After centrifugation, the supernatant was obtained for measurement by fluorescence spectrophotometer. The linear regression had a coefficient of estimation (r^2) at least 0.999 at the concentration ranging from 0.12 μg mL^{-1} to 7.8 μg mL^{-1}. The average recovery was 53.0% ± 1.1%.

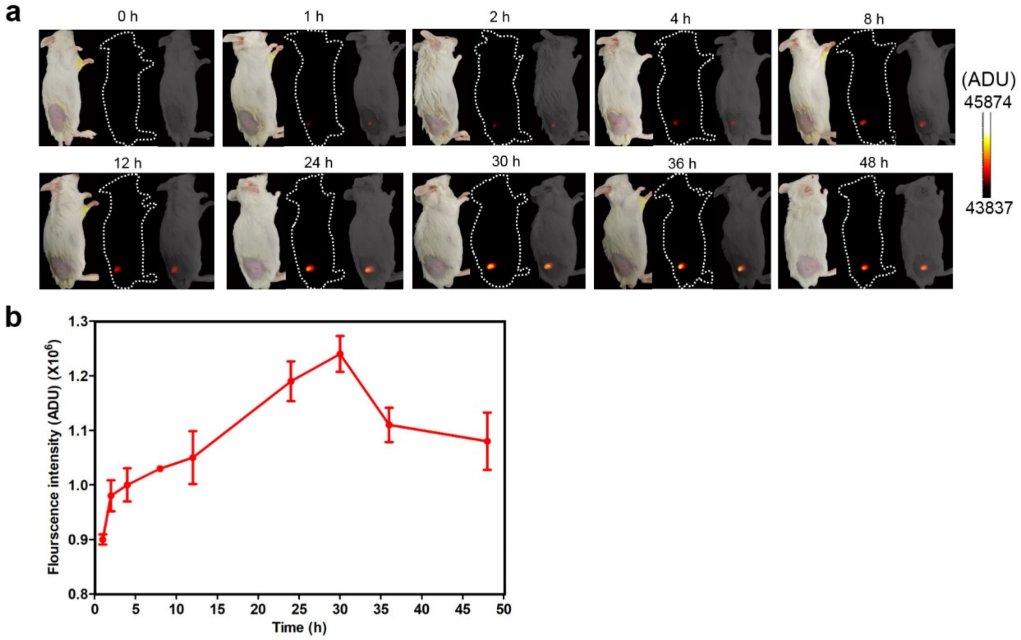

Supplementary Figure 9 NIR-II fluorescence live imaging of subcutaneously inoculated CT26-Luc tumor in BALB/c mice. (**a**), Light and NIR-II fluorescence images before (0 h) or at different time after i.v. injection of BPBBT NPs (20 mg kg^{-1}). (**b**), NIR-II fluorescence intensity of the tumor area over time. Data are presented as Mean ± S.D. (n = 3).

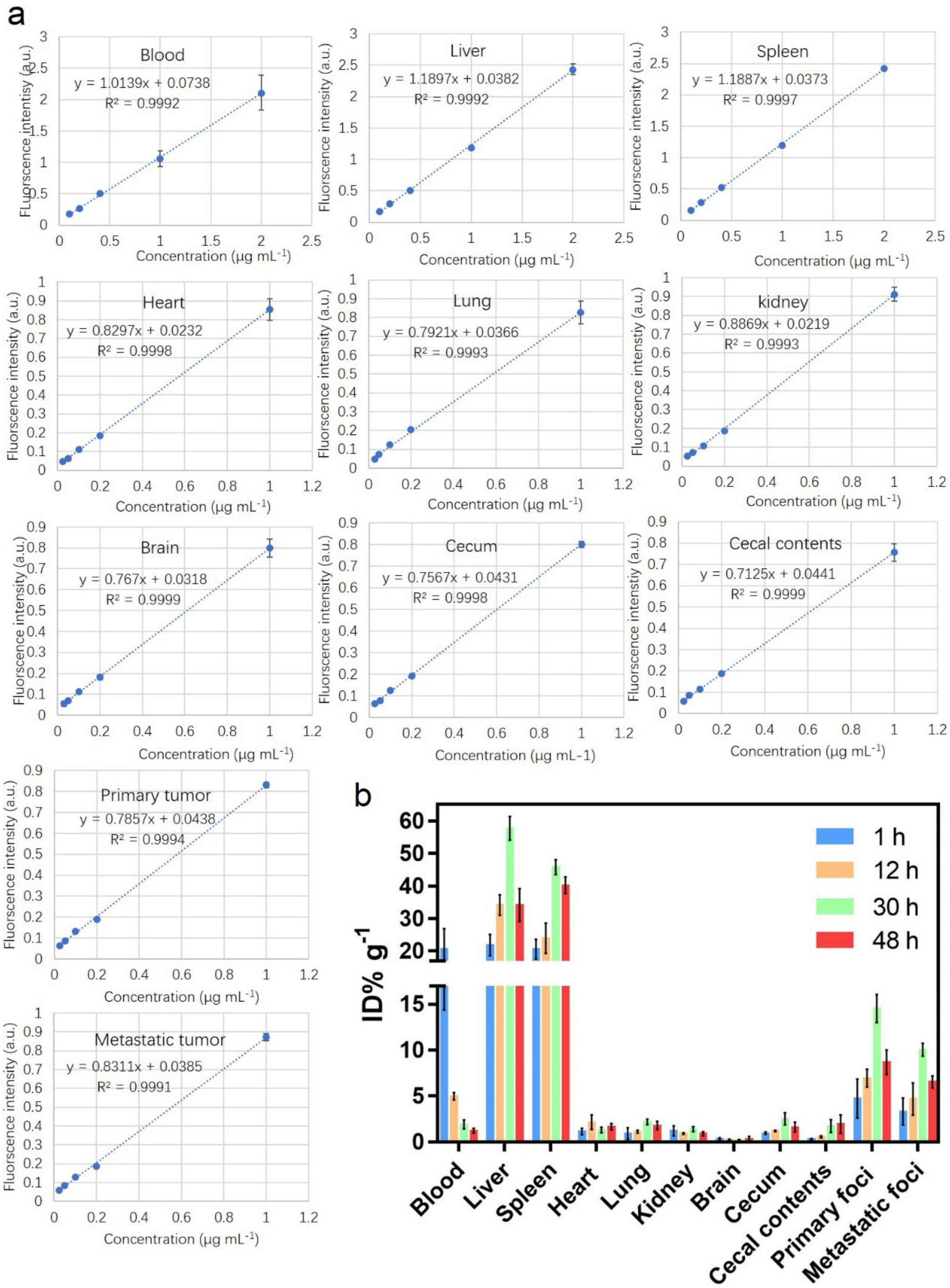

Supplementary Figure 10 (**a**), Linearity and calibration curves of BPBBT in different organs or blood ($n = 3$). (**b**), Biodistribution profile of BPBBT in CT26-Luc orthotopic tumor-bearing mice at various time intervals (1, 12, 30 or 48 h) after i.v. injection of BPBBT NPs (20 mg kg^{-1}) ($n = 5$). The amount of BPBBT was determined by measurement of its fluorescence intensity extracted from the tissue. ID% g^{-1}, Percentage of the injected dose per gram tissue. Data are presented as Mean ± S.D.

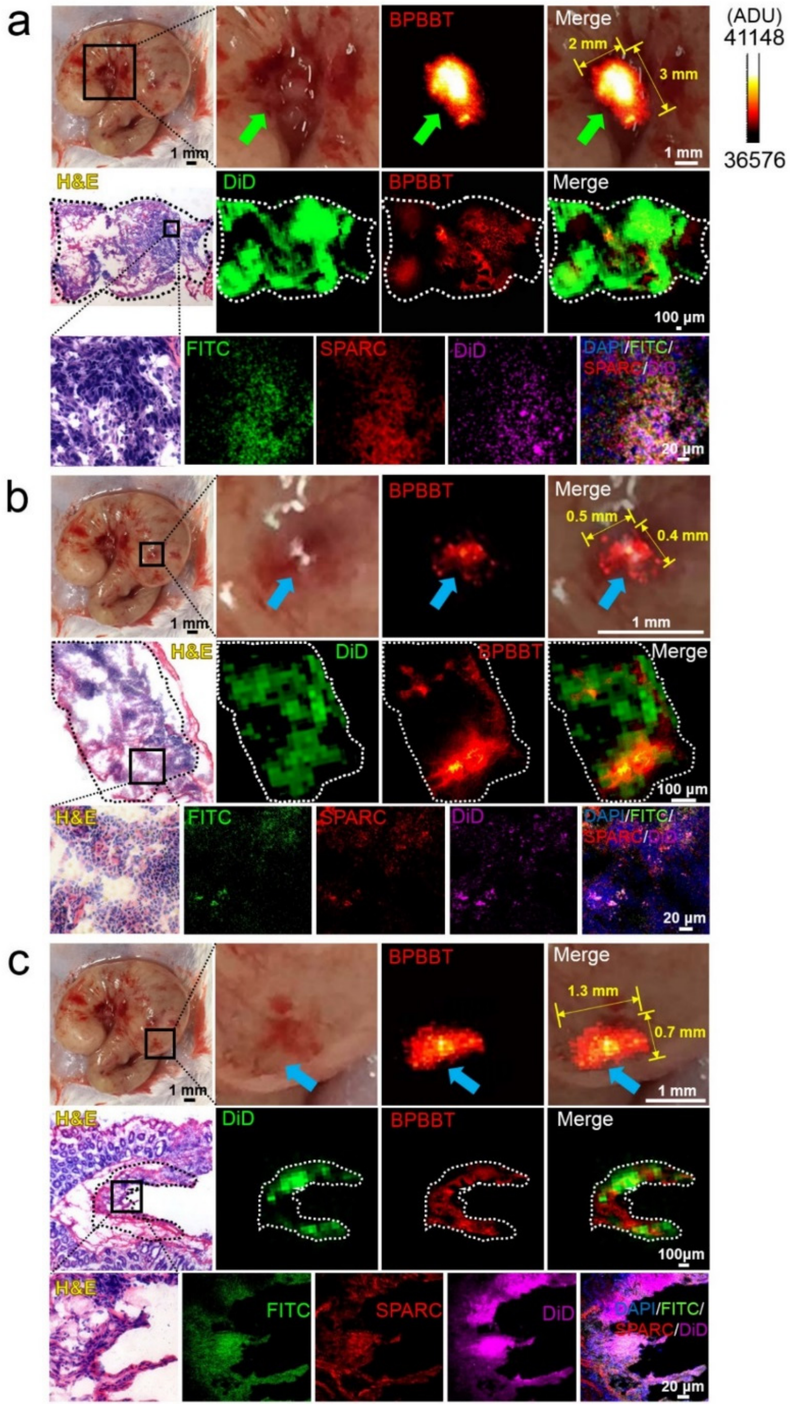

Supplementary Figure 11 Intraoperative NIR-II imaging of mouse 2 bearing orthotopic CT26 colon cancer by BPBBT NPs. BALB/c mouse bearing DiD-labeled CT26-Luc tumor was i.v. injected with BPBBT NPs (20 mg kg^{-1}). (**a**), NIR-II live imaging (upper) and histologic analysis (middle and lower) of primary tumor in the cecum of mouse at 30 h following the injection. Green arrows, primary tumor. (**b, c**), NIR-II live imaging (upper) and histologic analysis (middle and lower) of metastatic tumor foci in 0.5 mm × 0.4 mm size (**b**) or in 1.3 mm × 0.7 mm size (**c**) in the cecum. Blue arrows, metastatic lesions. Dashed circles, tumor area.

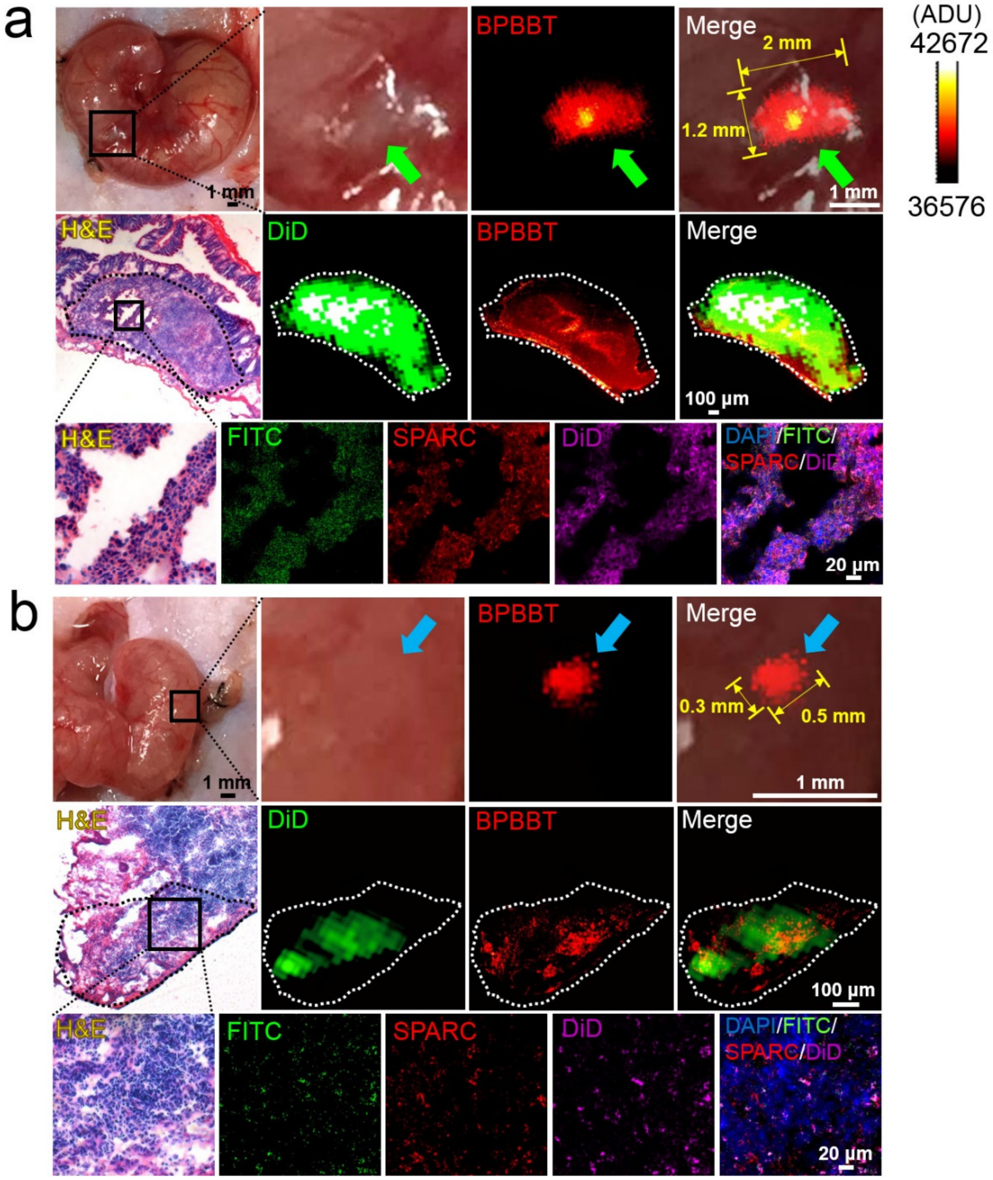

Supplementary Figure 12 Intraoperative NIR-II imaging of mouse 3 bearing orthotopic CT26 colon cancer by BPBBT NPs. BALB/c mouse bearing DiD-labeled CT26-Luc tumor was i.v. injected with BPBBT NPs (20 mg kg^{-1}). (**a**), NIR-II live imaging (upper) and histologic analysis (middle and lower) of primary tumor in the cecum of mouse at 30 h following the injection. Green arrows, primary tumor. (**b**), NIR-II live imaging (upper) and histologic analysis (middle and lower) of metastatic lesion (blue arrows) in 0.5 mm × 0.3 mm size in the cecum. Dashed circles, tumor area.

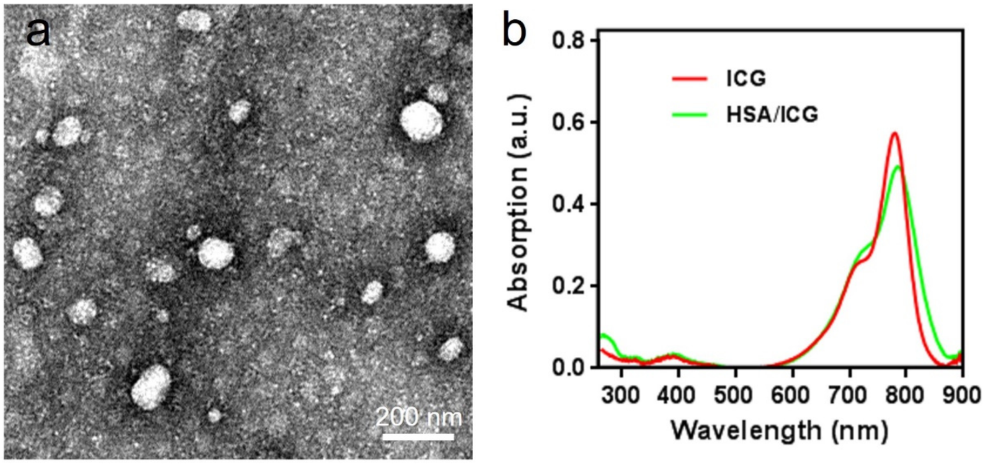

Supplementary Figure 13 (**a**), TEM image of HSA/ICG complexes. (**b**), UV-Visible absorption spectra of ICG (10 μM) and HSA/ICG complexes (10 μM of ICG) in water, respectively.

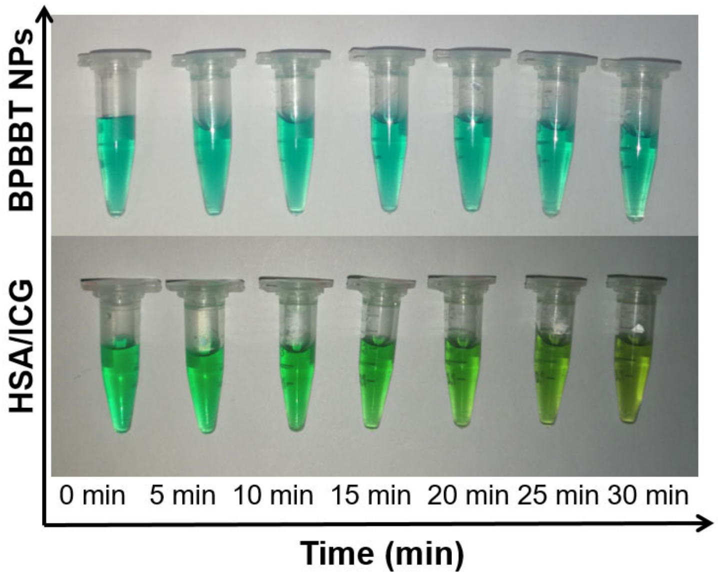

Supplementary Figure 14 Photographs of BPBBT NPs (200 μM of BPBBT) or HSA/ICG complexes (200 μM of ICG) in PBS solution before (0 min) or at different time after laser irradiation (808 nm, 0.8 W cm^{-2}).

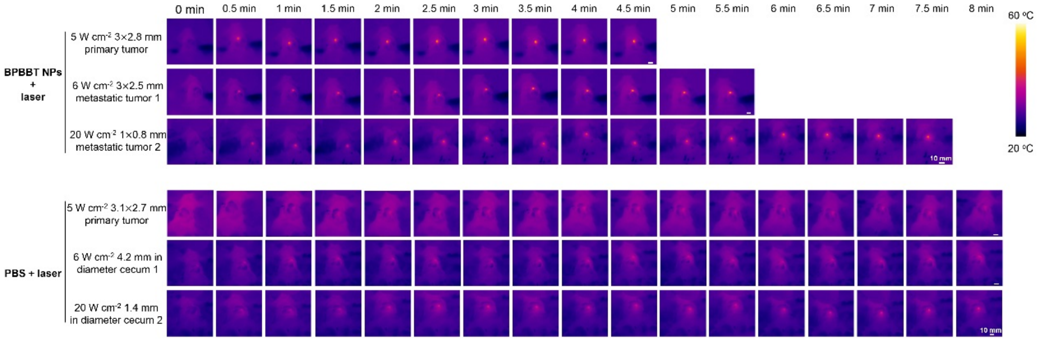

Supplementary Figure 15 IR thermal images before (0 min) and at different time points following PTT of the tumor bearing mice at 30 h following i.v. injection with BPBBT NPs (20 mg kg^{-1}) or PBS. In the BPBBT NPs-treated mice, primary and metastatic tumor was identified under NIR-II imaging followed by PTT with the indicated laser power density. In the PBS group, primary tumor was visualized by eye. Due to the invisible metastatic lesions, areas of cecum adjacent to the primary lesion were randomly selected for PTT.

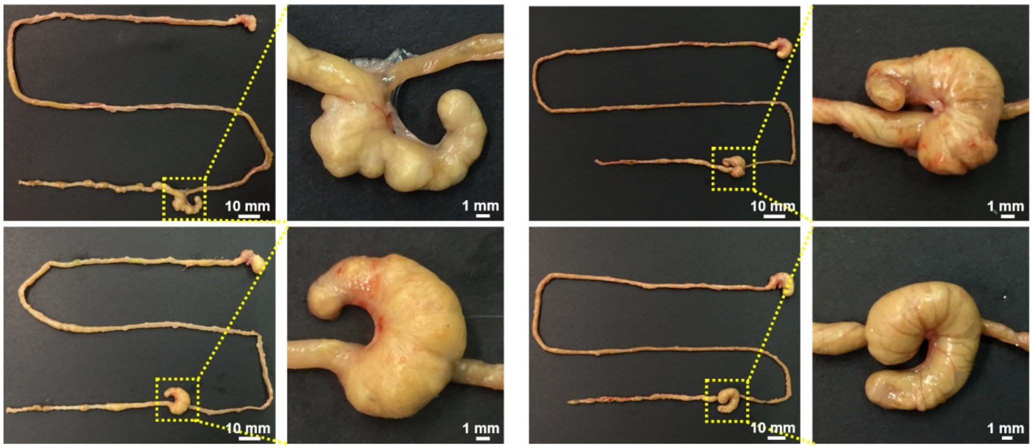

Supplementary Figure 16 Photographs of the resected gastrointestinal tract including cecum of the other four mice in NIR-II image-guided PTT group at 30 d following the treatment.

Supplementary Table 1. Blood chemistry and hematologic analysis of mice[a]

Item	Reference range	Control	Day 1	Day 7	Day 14	Day 30
WBC (10^9 L^{-1})	4.58-16.21	5.6 ± 0.8	4.1 ± 1.1	4.6 ± 1.3	3.1 ± 1.0	4.4 ± 1.1
LYMPH (10^9 L^{-1})	2.68-11.34	3.7 ± 0.4	3.1 ± 0.9	3.4 ± 1.0	2.4 ± 0.8	3.6 ± 1.0
MONO (10^9 L^{-1})	0.22-1.49	0.2 ± 0.1	0.1 ± 0.0	0.2 ± 0.1	0.1 ± 0.0	0.1 ± 0.0
NEUT (10^9 L^{-1})	0.54-4.31	1.5 ± 0.1	0.9 ± 0.2	1.0 ± 0.2	0.6 ± 0.1	0.7 ± 0.1
LYMPH (%)	43.45-86.46	68.7 ± 2.5	75.9 ± 1.5	73.5 ± 3.9	76.7 ± 1.9	81.5 ± 1.5
MONO (%)	3.40-13.44	3.8 ± 0.7	2.7 ± 0.3	3.1 ± 0.4	3.0 ± 0.5	2.4 ± 0.7
NEUT (%)	7.30-41.77	30.1 ± 2.6	21.4 ± 1.2	23.4 ± 4.0	20.4 ± 1.6	16.1 ± 1.5
RBC (10^{12} L^{-1})	7.17-11.35	7.4 ± 0.6	8.1 ± 0.4	8.2 ± 0.7	8.3 ± 0.2	8.1 ± 0.3
HGB (g dL^{-1})	11.2-17.8	11.1 ± 1.3	12.0 ± 0.5	12.2 ± 0.8	11.9 ± 0.3	12.5 ± 0.5
HCT (%)	38.2-64.0	36.6 ± 4.0	40.2 ± 1.4	39.9 ± 3.0	41.5 ± 0.8	39.2 ± 1.6
MCV (fL)	47.5-66.7	49.5 ± 1.7	50.0 ± 1.4	49.1 ± 0.7	50.3 ± 1.0	48.1 ± 0.6
MCH (pg)	12.9-18.1	14.9 ± 0.7	14.8 ± 0.2	15.0 ± 0.3	14.4 ± 0.4	15.2 ± 0.3
MCHC (g dL^{-1})	21.9-33.5	30.1 ± 0.4	29.8 ± 0.6	30.6 ± 0.3	28.7 ± 0.7	31.8 ± 0.4
RDW (%)	14.7-19.1	16.6 ± 0.9	16.4 ± 0.6	17.5 ± 0.2	16.9 ± 0.2	15.3 ± 0.4
PLT (10^9 L^{-1})	469-2364	617.3 ± 29.3	694.3 ± 67.9	559.0 ± 101.6	1243.8 ± 74.9	766.5 ± 198.2
MPV (fL)	4.4-6.2	6.2 ± 0.1	5.6 ± 0.4	5.2 ± 0.1	5.5 ± 0.1	5.8 ± 0.1
BUN (mg dL^{-1})	9-24	23.9 ± 1.8	34.4 ± 3.5	34.1 ± 2.1	31.4 ± 4.4	23.3 ± 6.8
ALT (U L^{-1})	18-71	30.4 ± 9.7	28.6 ± 2.9	52.7 ± 8.9	36.4 ± 18.3	29.5 ± 6.9
AST (U L^{-1})	45-182	82.4 ± 25.4	79.1 ± 9.7	137.3 ± 56.5	75.4 ± 14.4	78.6 ± 20.9

[a)] Blood or serum sample were collected from ICR mice (female, 4-6 weeks, 18-22 g) at 1, 7, 14 or 30 d after i.v. injection of BPBBT NPs (20 mg kg^{-1} of BPBBT). Mice without treatment were used as Control. Complete blood counts: Blood levels of White blood cells (WBC), Lymphocytes (LYMPH), Monocytes (MONO), Neutrophils (NEUT), Red blood cells (RBC), Hemoglobin (HGB), Hematocrit (HCT), Mean corpuscular volume (MCV), Mean corpuscular hemoglobin (MCH), Mean corpuscular hemoglobin concentration (MCHC), Red cell volume distribution width (RDW), Platelets (PLT), Mean platelet volume (MPV). Serum biochemistry data including blood urea nitrogen (BUN) levels and liver function markers such as Glutamic-pyruvic transaminase/ALT and Aspartate aminotransferase (AST) were also measured. Reference ranges of hematology data of healthy female ICR mice were obtained from Charles River Laboratories: (http://www.criver.com/). Data are presented as mean ± S.D. (n = 4).

Supplementary References

1. Dai, Q*., et al.* Size-dependent composition and molar extinction coefficient of PbSe semiconductor nanocrystals. *ACS Nano* **3,** 1518-1524 (2009).
2. Roper, D. K., Ahn, W. & Hoepfner, M. Microscale Heat Transfer Transduced by Surface Plasmon Resonant Gold Nanoparticles. *J. Phys. Chem. C Nanomater. Interfaces* **111,** 3636-3641 (2007).
3. Sheng, Z*., et al.* Bright Aggregation-Induced-Emission Dots for Targeted Synergetic NIR-II Fluorescence and NIR-I Photoacoustic Imaging of Orthotopic Brain Tumors. *Adv. Mater.* **30,** e1800766 (2018).
