# Supplementary material for: Albumin tailoring fluorescence and photothermal conversion effect of near-infrared-II fluorophore with aggregation-induced emission characteristics
Source: Nat Commun. 2019 May 17;10:2206. doi: 10.1038/s41467-019-10056-9 (PMC6525245; doi:10.1038/s41467-019-10056-9)
Supplement: Supplementary file 3 — Description of Additional Supplementary Files [file 41467_2019_10056_MOESM3_ESM.pdf]

Description of Additional Supplementary Files

File Name: Supplementary Movie 1
Description: Intraoperative NIR-II fluorescence imaging of mouse 4 bearing orthotopic CT26 colon cancer at 30 h following intravenous injection of BPBBT NPs
